# Supplementary figures and images for: Integrating ecosystem benefits for sustainable water allocation in hydroeconomic modeling
Source: PLoS One. 2022 May 5;17(5):e0267439. doi: 10.1371/journal.pone.0267439 (PMC9070880; doi:10.1371/journal.pone.0267439)

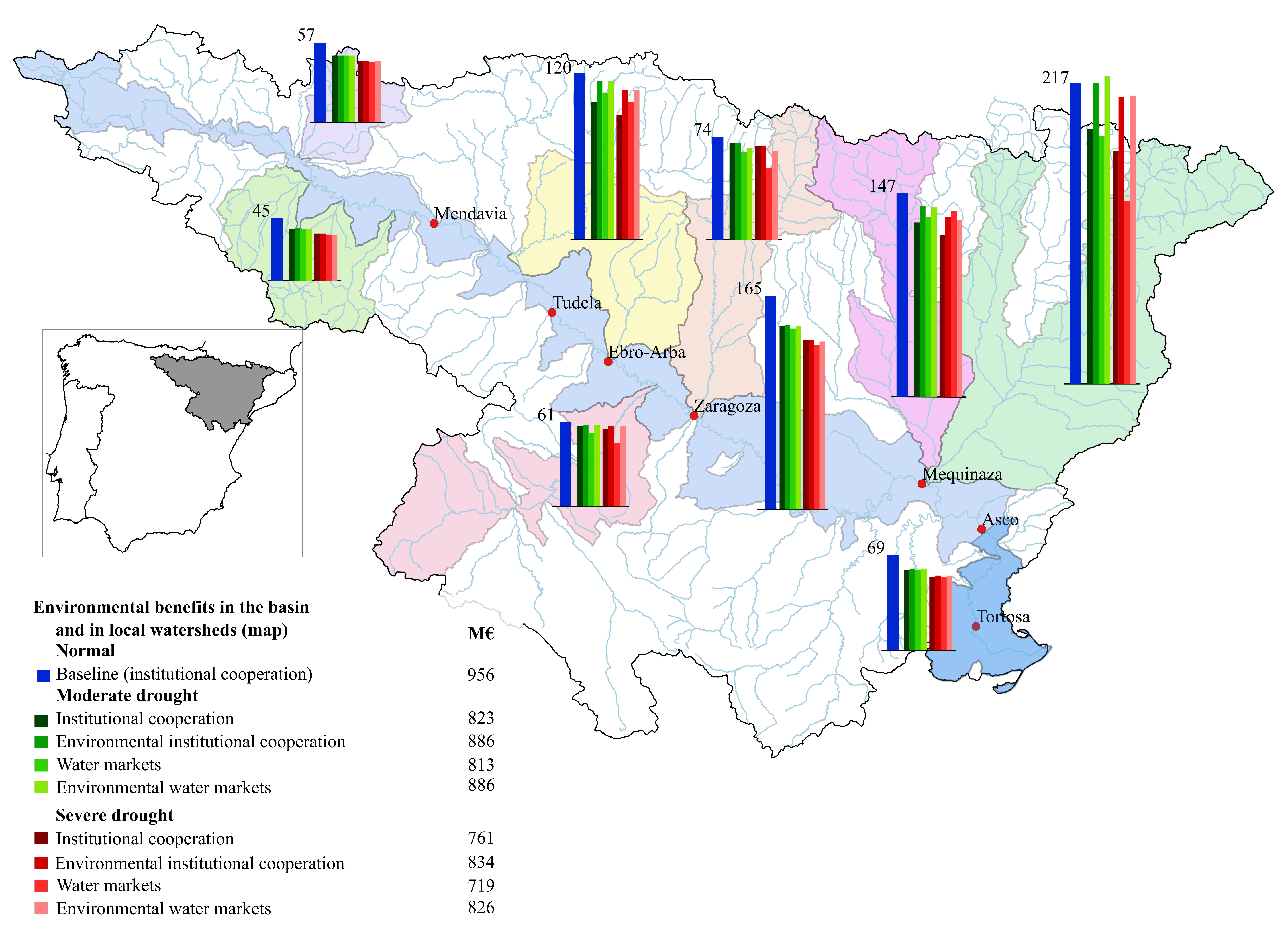

Supplement: S1 Fig — Reprinted from Confederación Hidrográfica del Ebro under a CC BY license, with permission from Confederación Hidrográfica del Ebro. a We present here additional results to the impacts of severe drought (-40% inflow reduction) considered in the main text of the article. These results correspond to a moderate drought scenario, where the reduction of inflows is lowered to 30% applying the same methodology. (JPG) [file pone.0267439.s001.jpg]

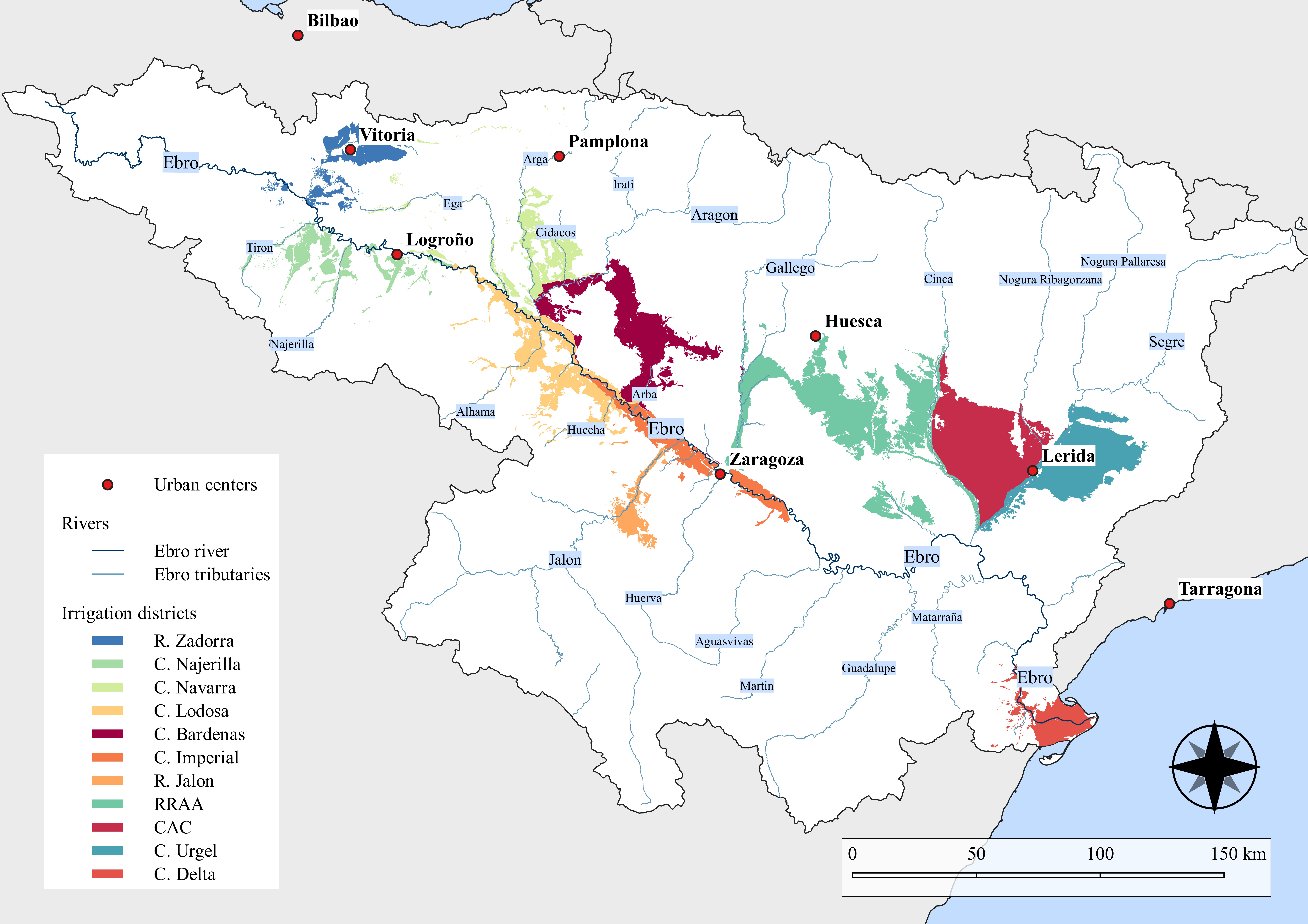

Supplement: S2 Fig — Reprinted from Confederación Hidrográfica del Ebro under a CC BY license, with permission from Confederación Hidrográfica del Ebro. (JPG) [file pone.0267439.s002.jpg]
