# Supplementary material for: Integrating ecosystem benefits for sustainable water allocation in hydroeconomic modeling
Source: PLoS One. 2022 May 5;17(5):e0267439. doi: 10.1371/journal.pone.0267439 (PMC9070880; doi:10.1371/journal.pone.0267439)
Supplement: S1 Table — a We present here additional results to the impacts of severe drought (-40% inflow reduction) considered in the main text of the article. These results correspond to a moderate drought scenario, where the reduction of inflows is lowered to 30% applying the same methodology. (DOCX) [file pone.0267439.s003.docx]

| Weather scenario | Normal | Moderate drought^a^ | | | | Severe drought^a^ | | | |
| --- | --- | --- | --- | --- | --- | --- | --- | --- | --- |
| Policy type | Base scenario | Institutional cooperation | Environmental institutional cooperation | Water markets | Environmental water markets | Institutional cooperation | Environmental institutional cooperation | Water markets | Environmental water markets |
| Water use (Mm^3^) | | | | | | | | | |
| Water use | 5,782 | 4,167 | 3,562 | 4,167 | 3,672 | 3,632 | 3,047 | 3,627 | 3,232 |
| Irrigation | 5,380 | 3,765 | 3,160 | 3,765 | 3,270 | 3,230 | 2,645 | 3,225 | 2,830 |
| Urban | 402 | 402 | 402 | 402 | 402 | 402 | 402 | 402 | 402 |
| Water exchanges |  |  | 605 | 435 | 820 |  | 585 | 235 | 780 |
| Between irrigation |  |  |  | 435 | 325 |  |  | 235 | 380 |
| Between irrigation and environment |  |  | 605 |  | 495 |  | 585 |  | 400 |
| Environmental flow in mouth | 8,895 | 6,230 | 6,440 | 6,220 | 6,370 | 5,350 | 5,540 | 5,345 | 5,435 |
| Irrigation surface area (1,000 ha) | | | | | | | | | |
| Surface area | 529 | 377 | 317 | 393 | 331 | 332 | 275 | 348 | 293 |
| Field crops | 400 | 260 | 204 | 272 | 217 | 219 | 165 | 229 | 182 |
| Fruit trees | 104 | 96 | 92 | 99 | 92 | 93 | 90 | 97 | 90 |
| Vegetables | 25 | 21 | 21 | 22 | 22 | 20 | 20 | 22 | 21 |
| Private and environmental benefits (€ M) | | | | | | | | | |
| Private benefit | 2,486 | 2,365 | 2,374 | 2,378 | 2,387 | 2,321 | 2,332 | 2,340 | 2,346 |
| Irrigation | 629 | 508 | 517 | 521 | 530 | 464 | 475 | 483 | 489 |
| Urban | 1,857 | 1,857 | 1,857 | 1857 | 1,857 | 1,857 | 1,857 | 1,857 | 1,857 |
| Environmental benefit | 956 | 823 | 886 | 813 | 886 | 761 | 834 | 719 | 826 |
| Social benefits | 3,442 | 3,188 | 3,199 | 3,191 | 3,211 | 3,082 | 3,105 | 3,059 | 3,118 |
