# Supplementary material for: Integrating ecosystem benefits for sustainable water allocation in hydroeconomic modeling
Source: PLoS One. 2022 May 5;17(5):e0267439. doi: 10.1371/journal.pone.0267439 (PMC9070880; doi:10.1371/journal.pone.0267439)
Supplement: Attachment — Submitted filename: PONE-D-21-31923_reviewer.pdf [file pone.0267439.s005.pdf]

PLOS ONE

Integrating ecosystem benefits for sustainable water allocation in hydroeconomic modeling

--Manuscript Draft--

**Manuscript Number:**	PONE-D-21-31923
**Article Type:**	Research Article
**Full Title:**	Integrating ecosystem benefits for sustainable water allocation in hydroeconomic modeling
**Short Title:**	Integrating ecosystem benefits in hydroeconomic modeling
**Corresponding Author:**	Jose Albiac, Ph.D.<br>University of Zaragoza<br>Zaragoza, SPAIN
**Keywords:**	Freshwater ecosystems; Droughts; Hydroeconomic modeling; Climate change; Water resources management
**Abstract:**	The increasing concern about the degradation of water-dependent ecosystems calls for considering ecosystems benefits in water management decision-making. Sustainable water management requires adequate economic and biophysical information on water systems supporting both human activities and natural ecosystems. This information is essential for assessing the social welfare of water allocation options. This paper evaluates various alternative water management policies by including the spatial and sectoral interrelationships between the economic and environmental uses of water. A hydroeconomic model is developed to analyze water management policies for adaptation to climate change in the Ebro Basin of Spain. The originality in our contribution is the integration of environmental benefits across the basin, by using biophysical information that relates stream flows and ecosystem status. The results show the enhancement of social welfare that can be achieved by protecting environmental flows, and the tradeoffs between economic and environmental benefits under alternative adaptation strategies. The introduction of water markets is a policy that maximizes the private benefits of economic activities, but disregards environmental benefits. The results show that the current institutional policy where stakeholders cooperate inside the water authority, provides lower private benefits but higher environmental benefits than markets, especially under severe droughts. However, the water authority is not allocating enough environmental flows to optimize social welfare. The protection of environmental flows in the Ebro Basin is a compelling strategic decision because of the imminent climate change impacts on water availability in coming decades.
**Order of Authors:**	Daniel Crespo
	Jose Albiac, Ph.D.
	Ariel Dinar
	Encarna Esteban
	Taher Kahil
**Additional Information:**	
**Question**	**Response**
**Financial Disclosure**<br><br>Enter a financial disclosure statement that describes the sources of funding for the work included in this submission. Review the submission guidelines for detailed requirements. View published research articles from *PLOS ONE* for specific	J.A received the funding from projects INIA RTA2014-00050-00-00 and INIA RTA2017-00082-00-00 of the Ministry for Science and Innovation, partly financed by European ERDF funds (https://www.ciencia.gob.es/site-web/).<br>The funders had no role in study design, data collection and analysis, decision to publish, or preparation of the manuscript.

Powered by Editorial Manager® and ProduXion Manager® from Aries Systems Corporation

examples.<br><br>This statement is required for submission and **will appear in the published article** if the submission is accepted. Please make sure it is accurate.<br><br>**Unfunded studies**<br>Enter: *The author(s) received no specific funding for this work.*<br><br>**Funded studies**<br>Enter a statement with the following details:<br>• Initials of the authors who received each award<br>• Grant numbers awarded to each author<br>• The full name of each funder<br>• URL of each funder website<br>• Did the sponsors or funders play any role in the study design, data collection and analysis, decision to publish, or preparation of the manuscript?<br>• **NO** - Include this sentence at the end of your statement: *The funders had no role in study design, data collection and analysis, decision to publish, or preparation of the manuscript.*<br>• **YES** - Specify the role(s) played.<br><br>* typeset	
**Competing Interests**<br><br>Use the instructions below to enter a competing interest statement for this submission. On behalf of all authors, disclose any competing interests that could be perceived to bias this work—acknowledging all financial support and any other relevant financial or non-financial competing interests.<br><br>This statement is required for submission and **will appear in the published article** if the submission is accepted. Please make sure it is accurate and that any funding sources listed in your Funding Information later in the submission form are also declared in your Financial Disclosure statement.	The authors have declared that no competing interests exist.

View published research articles from *PLOS ONE* for specific examples.<br><br>**NO authors have competing interests**<br><br>Enter: *The authors have declared that no competing interests exist.*<br><br>**Authors with competing interests**<br><br>Enter competing interest details beginning with this statement:<br><br>*I have read the journal's policy and the authors of this manuscript have the following competing interests: [insert competing interests here]*<br><br>* typeset	
**Ethics Statement**<br><br>Enter an ethics statement for this submission. This statement is required if the study involved:<br><br>• Human participants<br>• Human specimens or tissue<br>• Vertebrate animals or cephalopods<br>• Vertebrate embryos or tissues<br>• Field research<br><br>Write "N/A" if the submission does not require an ethics statement.<br><br>General guidance is provided below. Consult the submission guidelines for detailed instructions. **Make sure that all information entered here is included in the Methods section of the manuscript.**	N/A

**Format for specific study types**<br><br>**Human Subject Research (involving human participants and/or tissue)**<br>• Give the name of the institutional review board or ethics committee that approved the study<br>• Include the approval number and/or a statement indicating approval of this research<br>• Indicate the form of consent obtained (written/oral) or the reason that consent was not obtained (e.g. the data were analyzed anonymously)<br><br>**Animal Research (involving vertebrate animals, embryos or tissues)**<br>• Provide the name of the Institutional Animal Care and Use Committee (IACUC) or other relevant ethics board that reviewed the study protocol, and indicate whether they approved this research or granted a formal waiver of ethical approval<br>• Include an approval number if one was obtained<br>• If the study involved *non-human primates*, add *additional details* about animal welfare and steps taken to ameliorate suffering<br>• If anesthesia, euthanasia, or any kind of animal sacrifice is part of the study, include briefly which substances and/or methods were applied<br><br>**Field Research**<br><br>Include the following details if this study involves the collection of plant, animal, or other materials from a natural setting:<br>• Field permit number<br>• Name of the institution or relevant body that granted permission	
**Data Availability**<br><br>Authors are required to make all data underlying the findings described fully available, without restriction, and from the time of publication. PLOS allows rare exceptions to address legal and ethical concerns. See the PLOS Data Policy and FAQ for detailed information.	Yes - all data are fully available without restriction

A Data Availability Statement describing where the data can be found is required at submission. Your answers to this question constitute the Data Availability Statement and **will be published in the article**, if accepted.<br><br>**Important:** Stating 'data available on request from the author' is not sufficient. If your data are only available upon request, select 'No' for the first question and explain your exceptional situation in the text box.<br><br>Do the authors confirm that all data underlying the findings described in their manuscript are fully available without restriction?	
**Describe where the data may be found in full sentences. If you are copying our sample text, replace any instances of XXX with the appropriate details.**<br><br>• If the data are **held or will be held in a public repository**, include URLs, accession numbers or DOIs. If this information will only be available after acceptance, indicate this by ticking the box below. For example: *All XXX files are available from the XXX database (accession number(s) XXX, XXX.).*<br>• If the data are all contained **within the manuscript and/or Supporting Information files**, enter the following: *All relevant data are within the manuscript and its Supporting Information files.*<br>• If neither of these applies but you are able to provide **details of access elsewhere**, with or without limitations, please do so. For example:<br><br>*Data cannot be shared publicly because of [XXX]. Data are available from the XXX Institutional Data Access / Ethics Committee (contact via XXX) for researchers who meet the criteria for access to confidential data.*<br><br>*The data underlying the results presented in the study are available from (include the name of the third party*	All relevant data are within the manuscript and its Supporting Information files.

and contact information or URL).<br>• This text is appropriate if the data are owned by a third party and authors do not have permission to share the data.<br><br>* typeset	
Additional data availability information:	

Powered by Editorial Manager® and ProduXion Manager® from Aries Systems Corporation

Title Page Click here to access/download;Manuscript;TitlePage.docx

Integrating ecosystem benefits for sustainable water allocation in hydroeconomic modeling

Daniel Crespo[1,3], José Albiac[2,3,4*], Ariel Dinar[5], Encarna Esteban[5,3], Taher Kahil[4]

[1] Agrifood Research and Technology Centre (CITA) and University of Zaragoza, Zaragoza. Spain

[2] Economics and Business School, University of Zaragoza, Spain

[3] Instituto Agroalimentario de Aragón (IA2), Zaragoza, Spain

[4] Water Security Research Group, Biodiversity and Natural Resources Program, International Institute for Applied System Analysis (IIASA), Laxenburg, Austria

[5] School of Public Policy, University of California, Riverside, United States of America

[6] School of Humanities and Social Sciences, University of Zaragoza, Teruel, Spain

[*]Corresponding author

E-mail: maella@unizar.es (JA)

[¶]These authors contributed equally to this work.

Manuscript Click here to access/download;Manuscript;Manuscript.docx

Abstract

The increasing concern about the degradation of water-dependent ecosystems calls for considering ecosystems benefits in water management decision-making. Sustainable water management requires adequate economic and biophysical information on water systems supporting both human activities and natural ecosystems. This information is essential for assessing the social welfare of water allocation options. This paper evaluates various alternative water management policies by including the spatial and sectoral interrelationships between the economic and environmental uses of water. A hydroeconomic model is developed to analyze water management policies for adaptation to climate change in the Ebro Basin of Spain. The originality in our contribution is the integration of environmental benefits across the basin, by using biophysical information that relates stream flows and ecosystem status. The results show the enhancement of social welfare that can be achieved by protecting environmental flows, and the tradeoffs between economic and environmental benefits under alternative adaptation strategies. The introduction of water markets is a policy that maximizes the private benefits of economic activities, but disregards environmental benefits. The results show that the current institutional policy where stakeholders cooperate inside the water authority, provides lower private benefits but higher environmental benefits than markets, especially under severe droughts. However, the water authority is not allocating enough environmental flows to optimize social welfare. The protection of environmental flows in the Ebro Basin is a compelling strategic decision because of the imminent climate change impacts on water availability in coming decades.

Keywords: Freshwater ecosystems, Droughts, Hydroeconomic modeling, Climate change, Water resources management

Introduction

Ongoing water management and policies across the world deal with water scarcity by reallocating water to the most financially profitable activities or to priority uses (e.g., drinking water), with little or no consideration for the effects of reallocations on aquatic ecosystems. This inadequate recognition of the environmental services and related benefits in water allocation decisions has resulted in the degradation of many valuable ecosystems globally. In fact, biodiversity in inland aquatic ecosystems is the most threatened of all ecosystems [1,2]. Overuse of water resources and ecosystem status deterioration have led to a decline in the environmental services provided by ecosystems [3]

The growing concern about the environment fosters new methodologies for assessing environmental impacts of water degradation and improve policy decision making. Sustainable water management includes the environmental externalities in water allocation decisions, although the complex response of ecosystems to changes in environmental flows and imprecise environmental valuation make it difficult to identify optimal environmental flow requirements.

The expanding withdrawals by economic activities and the declining water availability as a result of droughts and the impending climate change are worsening water scarcity problems in arid and semi-arid regions. Environmental flows must remain at sufficient levels to protect ecosystem health, although this will reduce the water available for economic sectors. The sustainable management of water resources needs scientific knowledge and appropriate information for enhancing the balance between human water withdrawals and environmental flows in basins. Freshwater ecosystems provide goods and services to society, which have characteristics of public good or common pool

resources. Sustainable water allocation requires identifying and valuing the benefits of environmental services along with the private benefits of economic activities. Current water policies mostly ignore the public good and common pool aspects of environmental flows. Consequently, a more sustainable management must incorporate the external effects of human water withdrawals.

Hydroeconomic modeling is a valuable tool for identifying improved basin-level water management options. This tool integrates several aspects such as the spatial distribution of water resources and storage and transport infrastructures, water-based economic activities by sector and location, and water-dependent ecosystems. The advantage of hydroeconomic modeling is the linkage between hydrology, economy, environment, and institutions in evaluating water allocation alternatives. Hydroeconomic modeling could address a variety of problems, such as the analysis of water reallocation among sectors and locations, reservoir construction, interbasin transfers and hydropower production. The hydrology component represents the supply nodes of both surface and groundwater, and the demand nodes for irrigation, urban provision, and industrial and hydropower production.

Ecosystem protection in hydroeconomic models is usually represented by minimum environmental flows, because of the complexity of modeling the ecological response to streamflows. Some hydroeconomic models analyze environmental and also salinity damages in terms of water savings, replacement costs or crop production damages [4–8]. Recreation benefits such as boating and fishing are sometimes included in relation to streamflow levels, and travel cost or contingent valuation techniques are used for valuation of the ecosystem services [9–15]. Floods maintain the ecological status of riverine areas, and they preserve river ecosystem health because riverine areas provide critical functions like food supply and refugee to species. Floods and droughts' length

are included in hydroeconomic models assuming nonlinear responses between environmental costs and length of droughts.

Estimating environmental benefits or damages in riverine areas is difficult because of lack of information, so proxies are used such as irrigation benefits by Grafton et al. [16]. A better option is to analyze the dependence between wetland area and streamflow, and then select values for environmental services per unit of wetland area from valuation studies [17–20]. However these environmental benefit functions are not based on biophysical processes since the response of ecosystems to stream flows across river reaches is mostly unknown. Very few hydroeconomic studies specify ecological responses based on biophysical principles. Yang and Cai [21] include fish diversity in a multiobjective optimization problem using the Shannon index. Bryan et al. [22] undertake a more extensive approach by considering the aggregated response of birds, vegetation and fish based on biophysical information.

In any case, the representation of environmental benefits in hydroeconomic modeling is patchy and limited. The reason is the insufficient knowledge on the relationships between physical, ecological, and valuation variables, and the uncertainty on critical environmental thresholds [23]. The scope of the environmental benefits is usually limited to a small area like a wetland, lake or river reach, and the range of spatial interactions is narrow. In order to overcome these limitations, we estimate environmental benefits based on biophysical processes covering most river reaches in the whole basin.

The contribution of this paper over previous literature is to include the benefits of environmental flows supporting ecosystems in decision making. This is an advance in hydroeconomic modeling that has not been fully developed, because of the difficulties in incorporating the environmental components. The innovation over previous

hydroeconomic modeling is the calculation of environmental benefits in river reaches, using biophysical information that relates stream flows and ecosystem status. Then, the environmental benefits of river reaches are integrated at basin scale in a framework that accounts for the spatial and sectorial tradeoffs of water allocation, including environmental flows.

The benefits of ecosystem services can be estimated by finding the response of ecosystems to water flows, and then valuating the services provided by these ecosystems. Information on environmental benefits in hydroeconomic models is quite limited because of the difficulties in identifying ecosystems and their services, and how these respond to changes in stream flows. Several studies include ecosystem water consumption in hydroeconomic modeling [24,25], however the undetermined response of ecosystems to environmental flows and the scarcity of valuation estimates undermine the accuracy of results. A common procedure for unknown ecosystem benefits in hydroeconomic modeling and also in pragmatic water management is to set up minimum environmental flows.

The analysis in this paper focuses on the economic and environmental impacts of droughts and water scarcity in the Ebro basin, and the social welfare that can be achieved under alternative water allocation policies. The analysis is based on the development of a hydroeconomic model with three components: a reduced hydrological component, a regional economy component, and an environmental component. The economy component includes the main urban and irrigation water uses, and the environmental component includes the ecosystem health and the associated environmental benefits. The novelty of this paper lies in the modeling of the environmental component, in which the ecological status response to stream flows is represented using information from biophysical studies relating the flow in river tracts

with ecosystem health. The ecological status is an indicator of the potential of ecosystems to provide goods and services, and it proxies the environmental benefits received by society.

Selected water allocation policies have been evaluated for different water availability conditions in the basin. The water allocation policies considered are: i) the current institutional cooperation, based in proportional allocation between irrigation districts; ii) environmental institutional cooperation, where environmental flows are increased to maximize social welfare; iii) water markets, which maximize private benefits; and iv) environmental water markets, where users and the environment exchange water to maximize social welfare. This policy selection follows the approach suggested by Kahil et al. [26]. Institutional cooperation is the current allocation mechanism based on collective action by stakeholders, rather than on administrative coercion or monetary incentives (e.g. pricing), where water allocation decisions are taken by stakeholders' groups at both basin and local watershed board levels.

The paper is structured as follows. Next section explains the methodology, describing the linkage between ecosystem status and environmental benefits, the response of ecosystems to stream flows, the Ebro basin and the hydroeconomic model framework, and the policy scenarios. Section three presents the results and discussion, and section four concludes with the main findings and policy implications.

Methods

Ecosystem status and environmental benefits

Interactions between economic activities and the health of ecosystems are subject to multiple and complex biophysical processes, and these interactions play an important

role in determining the impact of economic activities on nature [23]. The impacts are strong, changing ecosystems habitats and their provision of environmental services. Harmful alterations of biophysical conditions in ecosystems degrade their functioning and processes, diminishing the services and benefits provided to humans [27].

Sustainable water management policies recognize the environmental contribution to human wellbeing. Modeling the relationship between environmental benefits and stream flows is a challenging task. Environmental benefits are obtained from the biophysical conditions of ecosystems and the economic valuation of the services provided (Fig 1). Both the ecological response to biophysical conditions and the valuation of ecosystem goods and services need to be determined for an adequate representation of environmental benefits [28].

Fig 1 Environmental benefit diagram

Alterations in the hydrological regime and ecological responses

Hydrological regime alterations lead to changes in ecological response. The most common cause of hydrological alterations is water withdrawals for irrigation, urban and industrial use, the construction of levees to control flooding, and the construction of dams and reservoirs [29]. These activities cause hydrologic alterations: withdrawals reduce stream flows in basins, levees modify the morphology of the river channel changing water speed, hydrological cycles, and flooding intensity and frequency [30–33]. Consequently, the ecological response of ecosystems is determined by the type of alteration created. Studies analyzing the ecological response focus on specific types of hydrological regime alterations. These studies usually investigate the response of a specie or set of species to an alteration in the hydrological regime. The response is defined by an indicator of the health of ecosystems. These analyses examine the

response at population, community, and ecosystem levels [29], although population and community indicators are only partial as they do not show the health status of the entire ecosystem[34].

Population indicators in ecological response studies are based on variables such as the number of individuals, size and weight, mating frequency and the presence or absence of a specie of interest. Community and ecosystem indicators emphasize the importance of biodiversity, and underline the quality and diversity of ecosystems. The indicators for analyzing biodiversity determine the extent to which ecosystems have been homogenized as a consequence of alterations in the hydrological regime [35].

Determining and estimating the functional form of the ecological response are challenging tasks [36]. Statistical estimation, classification and prediction techniques of the ecosystem status range from simple methods such as econometric regression, principal component analysis and non-parametric estimation methods, to more sophisticated methods such as random forests and neural networks [37–40].

Ecosystem functions, services and values

Ecological processes and ecosystem structure and function are strongly related to flow and hydrological variations which drive the ecological status [41]. Many ecological processes are flow-dependent, although information on the response of ecosystems to flow alterations is limited. Ecological responses to flow variations affect invertebrate populations, fish and riparian vegetation, and human alterations are usually negative [29]. The functions and processes of ecosystems generate services to society, but degradation of the ecological status deteriorates these services. Altering the hydrological regime sets off a chain of negative effects on ecosystem status and benefits for society [42].

Ecosystem services are defined as the benefits of nature to households, communities, and economies [43], or else as the ecosystem functions and processes that humans benefit from directly and indirectly [44]. There are several classifications of ecosystem services based on advances in knowledge, progress in the economic valuation of the environment, and emerging interests in the policy process. The classifications are similar and are grouped into four categories: provision, regulation, habitat or medium, and culture and recreation [1,44–46].

Goods and services from ecosystems provide society with environmental benefits which mostly show public good or common pool characteristics, so they are market externalities in the decision-making concerning economic activities. The balance between environmental and private benefits should be considered for a more sustainable water management, so that water allocations are driven not only by market activities but also by protection of ecosystems. Consequently, both the environmental and private benefits of water have to be recognized in sustainable water allocations.

Information on the valuation of ecosystems' goods and services is needed for calculating environmental benefits, but the specific values of ecosystem services by type and location remain an unsettled question [47]. Economic valuations are mostly grounded on revealed or stated willingness to pay approaches, based on individual preferences. The contribution of ecosystem services to wellbeing may not be well assessed by these estimations, and valuation results are quite disparate and largely debated. However, total economic valuation from use and no-use values is the approach broadly accepted to estimate the value of ecosystems [47].

Modeling the response of ecosystems to stream flows

Modeling environmental benefits requires identifying the relationship between flow regime, ecosystem status, and the value of ecosystem services. Studies on environmental flow assessment contribute to water management by providing information about the link between changes in flow regime and the consequences for the environment. Instream flow methods based on physical habitat simulation, as wetted perimeter and weighted usable area (WUA), are frequently included in environmental flow studies. This methodology accounts for the hydrological, hydraulic (hydraulics involves physical and mechanical properties such as water velocity and water depth), and biological relationships in order to evaluate environmental flow requirements [48].

WUA is used for the assessment of environmental flows in several river basins, but also in studies analyzing management policies for protection of stream flows and ecological status [49,50]. Ecosystem habitat and biodiversity are indicators of the ecosystem status, which are used in defining the relationship between flow regime and ecosystem health. Then, environmental benefits are the economic valuation of services corresponding to the ecosystem health level. Several institutions and projects support the valuation of ecosystem services related to water resources, by providing and collecting valuation studies.

Modeling environmental response and ecosystem status

Habitat quantity and quality are essential for ensuring that ecosystems status and biodiversity remain healthy. Consequently, the relationship between habitat and flow determines the ecological potential in rivers. The wetted perimeter in a river is a fast and simple way to measure the size of the habitat in a river section. This variable is the river surface area covered by water. Other more advanced methods such as the WUA

combine organism preferences and physical variables in the river section, resulting in the characterization of the potential habitat.

The WUA is based on land studies that collate physical information on different river reaches. Because these land studies are costly and require dedicated resources, their use is limited. The data obtained from field studies help simulate the velocity and depth of the water that is generated by a specific flow. Simulations of water velocity and depth, riverbed composition and fish preferences are variables used for assessing the relationship between flow and habitat size.

WUA studies provide information on habitat potential in a river tract for a species. The streamflow determines habitat size, which in turn determines ecosystem health status. The WUA is a proxy for ecosystem status indicating ecosystem health. Then, a functional form can be selected to represent the response of habitat to flow variables.

Environmental response function: the habitat-flow function

Setting up environmental flows in river tracts is burdensome because of the difficulties in obtaining habitat-flow relationships with costly field studies. This is solved by using methods that extrapolate the habitat-flow relationship to other parts of the river. These methods estimate the parameters from a functional approach that links WUA and flow. Many functional forms are used in these methods, for example quadratic functions and exponential functions [51,52]. Based on functions used in these studies, the functional form chosen to characterise ecosystem status is the following exponential form:

$$WUA_s(X_s) = 1 - e^{\beta_s X_i}$$

where WUA_s is the weighted usable area in river section s, X_s is the flow in the section, and β_s is the parameter characterizing the WUA response in section s.

This function provides an index of the health status of ecosystems. Parameter α must be negative and the function meets the following conditions: the values of the function range from zero to one; the function is strictly increasing; and *WUA* is zero when flow is zero and approaches one as flow rises.

The Ebro basin

The Ebro basin is located in the north-east of the Iberian Peninsula. It covers an area of 85,600 km^2 and supports the economic activities of 3.2 million inhabitants. Renewable water resources amount to 14,600 million cubic meters (Mm^3) per year. Water withdrawals total 8,460 Mm^3, of which 8,110 Mm^3 are surface water diversions and 350 Mm^3 groundwater extractions [53]. Water withdrawals for agriculture are 7,680 Mm^3 covering 700,000 hectares of irrigated crops. Water abstractions in urban systems amount to 630 Mm^3 and direct industry abstractions are nearly 150 Mm^3. Non-consumptive water withdrawals are used for cooling thermal power plants (3,100 Mm^3) and for hydropower production (38,000 Mm^3). Water for agriculture represents 90% of consumptive water demand, and the main irrigated crops are corn, barley, alfalfa, wheat and fruit trees.

Red Natura 2000 spaces are special protection areas for habitat and species. There are 374 Red Natura 2000 spaces totaling an area of nearly 25,500 km^2 in the Ebro basin. The number of water bodies that are either fully or partially in a Red Natura 2000 area is 664, amounting to 80% of all the water bodies in the basin [53]. Water management has been adapted to environmental targets and regulations governing protected areas. Protection in these areas is part of water plans, maintaining environmental flows even in exceptional conditions during droughts. Environmental flows are included in water planning to achieve the objective of good status in water bodies.

The Confederación Hidrográfica del Ebro (CHE—the Water Authority of the Ebro Basin) is the institution responsible for managing the water in the basin. A special characteristic of the institutional setting of water authorities in Spain is the crucial role played by user groups. Water management maintains the traditional culture of cooperation between organized groups of users, which characterizes water authorities. Users from every sector (irrigation, urban, industrial and hydropower), central and state governments, municipalities, farmers' unions, environmental associations, business associations and workers unions are represented in the water authority.

The CHE is responsible for preparing the water plan of the Ebro Basin, which sets out medium-term management strategies. The plan objectives are to cover water demand, contribute to regional development, and protect ecosystems in the basin. Ecosystems are protected by regulated environmental flows in each river reach. These environmental flows are minimum levels of stream flows that maintain the status of water-dependent ecosystems. In recent years, there has been a conflict between upstream and downstream states in the basin for the regulation of environmental flows at the Ebro mouth [54].

Environmental flows are based on information from hydrological studies and habitat studies of representative fish species. Hydrological studies analyze aspects such as flow seasonality, and the rate of change or river continuity. Habitat studies are weighted usable area studies relating the potential habitat for a species with the water flow, from which minimum environmental flows are selected. The Ebro Basin Authority has used a WUA study [53] covering 63 river reaches in order to define environmental flows for each river section. The study VANE [55] provides estimations of the value of environmental services in the Ebro basin, with an average value of 420 € per hectare.

This valuation includes services provided by rivers, lakes, wetlands, forests, agriculture and other habitats and land uses.

Modeling framework of the Ebro basin

The hydroeconomic model of the Ebro basin integrates hydrological, economic, environmental and institutional aspects. The model includes a reduced-form hydrological model, a regional economy component, and an environmental benefit component.

Reduced-form hydrological component

The hydrological component represents flows between supply and demand nodes using the hydrological principles of water mass balance and flow continuity in the river. The hydrological component shows the spatial distribution of water flows used by economic sectors and environmental flows. The mathematical formulation is as follows:

$$W_{out_d} = W_{in_d} - W_{loss_d} - Div_d^{IR} - Div_d^{URB} \tag{1}$$

$$W_{in_{d+1}} = W_{out_d} + r_d^{RI} \cdot (Div_d^{IR}) + r_d^{URB} \cdot (Div_d^{URB}) + RO_{d+1} \tag{2}$$

$$W_{out_{mouth}} \geq E_{mouth}^{min} \tag{3}$$

Equation (1) is the mass balance equation, and it determines water outflow W_{out_d} in river reach d, which is equal to water inflow W_{in_d}, minus water losses W_{loss_d}, water abstraction for irrigation Div_d^{IR}, and abstraction for urban and industrial use Div_d^{URB}. Equation (2) guarantees river flow continuity, in which water inflow in the following river reach $W_{in_{d+1}}$ is the sum of the water outflow from the previous reach W_{out_d}, return flows from previous irrigation districts $[r_d^{RI} \cdot (Div_d^{IR})]$, urban return flows $[r_d^{URB} \cdot (Div_d^{URB})]$, and the flow entering this river reach from tributaries RO_{d+1}. Equation (3)

states that water outflow at the mouth of the Ebro $W_{out_{mouth}}$ must be greater than or equal to the minimum environmental flow in the river reach.

The hydroeconomic model was calibrated, introducing auxiliary variables for every river reach so that the model replicates actual observations. Calibration is used to close the mass balance equation, since there are water inflows and outflows in the system that cannot be observed (for example, underground flows, evaporation or some return flows). Calibration includes non-observed flows, which are the difference between flows estimated with the model and flows measured at gauging stations.

Regional economic component

The regional economic component includes agricultural irrigation and urban water use. There is an optimization model for agricultural activities in every irrigation district, which maximizes farmers' private benefits from crop production subject to technical and resource constraints. Crop yield functions are assumed linear and decreasing, and output and input prices are constant. The optimization problem is formulated as follows:

$$Max(B_k^{IR}) = \sum_{ij} C'_{ijk} X_{ijk} \tag{4}$$

s.t.

$$\sum_i X_{ijk} \le T_{land_{kj}};\ j = flood, sprinkler, drip \tag{5}$$

$$\sum_{ij} W_{ijk} X_{ijk} <= T_{water_k} \tag{6}$$

$$\sum_{ij} M_{ijk} X_{ijk} \le T_{la_k} \tag{7}$$

$$X_{ijk} \ge 0 \tag{8}$$

where B_k^{IR} is private benefit in irrigation district k, and C'_{ijk} is net income of crop i using irrigation technology j. The decision variable of the problem is X_{ijk}, which is

acreage of crop i under irrigation technology j. Equation (5) represents the restriction of available land $T_{land_{kj}}$ in irrigation district k equipped with irrigation system j. The water available T_{water_k} in irrigation district k is given by equation (6), where W_{ijk} is the water requirement of crop i with technology j. The water available T_{water_k} is the variable linking the optimization model of irrigation districts and the hydrological component. The labor constraint (7) represents labor availability T_{la_k} in irrigation district k, where M_{ijk} is the labor requirement of crop i with irrigation system j.

This optimization model includes the major crops in every irrigation district. Irrigation systems for field crops are flood and sprinkler, and for fruit trees and vegetables are drip and flood. Net income per hectare C'_{ijk} is the difference between crop revenue and direct and indirect costs (including capital amortization) and it is expressed by $C'_{ijk} = P_i Y_{ijk} - CP_i$ where P_i is price of crop i, Y_{ijk} is yield of crop i under technology j in the irrigation district k, and CP_i are direct and indirect costs of crop i (including water costs).

The crop yield function is linear and represents a decreasing crop yield when additional land is assigned to crop production, based on the principle of Ricardian rent. The first lands in production have the highest yields, and yields fall off as less-suitable lands enter production. The crop function relates yields with acreage of crop i under irrigation technology j, and is defined as:

$$\mathrm{Y_{ijk}} = \beta_{0_{\mathrm{ijk}}} + \beta_{1\mathrm{ijk}}\mathrm{X_{ijk}} \quad (9)$$

The agricultural component is calibrated using Positive mathematical programming (PMP) to reproduce the observed land and water use under baseline conditions, and to address the problem of crop overspecialization [56]. Calibration follows the PMP

procedure by [57], where parameters are estimated for a linear yield function [Equation (9)] based on the first-order conditions of benefit maximization. Data on yields, prices, crop water requirements, production costs, availability of water resources, land and labor, together with information on biophysical parameters have been obtained from statistical databases, reports, previous studies and expert consultation [58-63].

The modeling of urban water maximizes economic surplus, the sum of consumer and producer surpluses in the basin's main cities. The optimization problem of the urban sector is expressed by:

$$MaxB_u^{URB} = (a_{du}Q_{du} - ½\, b_{du}Q_{du}^2 - a_{su}Q_{su} - ½\, b_{su}Q_{su}^2) \quad (10)$$

s.t.

$$Q_{du} - Q_{su} \leq 0 \quad (11)$$

$$Q_{du};\ Q_{su} \geq 0 \quad (12)$$

where B_u^{URB} is the consumer and producer surplus in city u. The variables Q_{su} and Q_{du} are water supply and demand in city u, respectively. The parameters a_{du} and b_{du} are the constant term and the slope of the inverse demand function, and the parameters a_{su} and b_{su} are the constant term and the slope of the water supply function. Equation (11) states that the supply must be equal to or greater than the demand for water. The water supply Q_{su} is the variable linking urban water with the hydrological component. The equation parameters have been obtained from the studies by Arbués et al. [64] and Arbués et al. [65].

Environmental component

The environmental benefits of aquatic ecosystem in the basin depend on the health status of ecosystems, where the relationship between the river's habitat status and

stream flows is expressed by the WUA. A study of the WUA in the Ebro Basin provides data for these relationships for every section in the basin, based on their hydrological characteristics. The relationships are estimated by an exponential function calibrated to the data from the WUA study [53].

The WUA study covers 14 segments of the hydrology network of the Ebro Basin, characterizing the ecological status in every section. The benefits aquatic ecosystems generate are given by the following expressions:

$$WUA_s(W_s) = 1 - e^{\beta_s W_s} \quad (13)$$

$$EW_s^{eco} = WUA_s(W_s) \quad (14)$$

$$B_s^{eco} = VE \cdot l_s \cdot EW_s^{eco} \quad (15)$$

where WUA_s is the weighted usable area section s, W_s is average flow in s, and EW_s^{eco} is the health status of ecosystem eco in s. The weighted usable area WUA_s depends on average flow W_s and the estimated parameter β_s, considering the months with less water availability. The parameter β_s has been estimated through non-linear regression for 14 of the 63 locations where information is available (Table 1).

WUA studies require collecting data of the river waterbed composition and undertake a topographic study of the river section. The information on the morphology and topology of the terrain enables the simulations of the hydraulic variables, water velocity and water depth, generated by a specific streamflow. This information is combined with habitat studies that describe fish preferences to water velocity, water depth and waterbed characteristics, in order to calculate the habitat potential. The WUA is the habitat potential weighted by the total area of the river section. The studies of the Ebro basin authority includes 64 river sections of the Ebro basin. For each river section,

Table 1. Results of the WUA regression for 14 sections of the Ebro.

$$WUA_{section} = 1 - e^{\beta_{section} * flow_{section}}$$

Section	*β*	*Standard error*	*t*	*Pr(>|t|)*	*Section*	*β*	*Standard error*	*t*	*Pr(>|t|)*
**ID.202**	-9.65	1.82	-5.31	< 0.01	**ID.428**	-0.30	0.03	-12.01	< 0.01
**ID.264**	-2.07	0.21	-9.84	< 0.01	**ID.433**	-0.10	0.01	-12.05	< 0.01
**ID.274**	-1.42	0.17	-8.52	< 0.01	**ID.441**	-8.14	2.75	-2.96	< 0.01
**ID.406**	-16.39	2.74	-5.98	< 0.01	**ID.446**	-1.96	0.31	-6.44	< 0.01
**ID.418**	-1.89	0.22	-8.74	< 0.01	**ID.455**	-9.61	2.53	-3.79	< 0.01
**ID.421**	-0.12	0.01	-13.54	< 0.01	**ID.463**	-0.23	0.05	-4.39	< 0.01
**ID.426**	-0.52	0.06	-7.95	< 0.01	**ID.662**	-0.26	0.02	-14.60	< 0.01

a representative fish species is selected and the WUA is obtained for three different life-stages: fray, juvenile and adult. WUA for a fish species for the dry and wet seasons are produced combining life-stage WUA results and the mean flow at the river section.

This relationship and its corresponding parameter for each river section determines the habitat size and the ecosystem potential to contain species given a specific flow. The benefit B_s^{eco} of ecosystems in section s depend on the environmental status EW_s^{eco}, the length l_s of the section, and the economic value VE_s of ecosystems' services. The estimated value of ecosystem services provided by rivers ranges from 1,450 to 35,600 € per hectare of the riverbed surface area covered by water [44,55,66,67].

Considering the extent of the range of estimated values in the literature and the value for basins similar to the Ebro Basin, three valuation levels are selected: low, medium and high environmental value. The low environmental value is 9,600 € per hectare for river sections with low environmental value in the main stem of the Ebro and in some right bank tributaries. The medium environmental value is 24,000 € per hectare for non-mountain Ebro tributaries. The high environmental value is 60,000 € per hectare for mountain river sections and also for the Ebro mouth where the Ebro Delta is located, which is of exceptional environmental value. The surface area of the rivers in the basin

covered by water is 68,000 ha, with a total length of 8,900 km [68]. The model only includes 4,700 km impacted by water withdrawals for economic activities.

Modeling policy scenarios

The optimization model integrates the hydrologic, economic and environmental components. The model maximizes the basin's benefits subject to hydrological, technical, resource and institutional constraints in every economic sector and location. The optimization problem is defined by the expression:

$$Max: \sum_k B_k^{ir} + \sum_u B_u^{urb} + \sum_s B_s^{eco} \quad (16)$$

subject to equations (1)– (15) and the constraints of water availability in the basin:

$$Div_d^l \leq W_{in_d} \quad \forall\ l, d \quad \text{where } l = k, u \quad (17)$$

$$\sum_d Div_d^l \leq \overline{W} \quad \forall\, l\ =\ k, u \quad (18)$$

where B_k^{ri} are the benefits of each irrigation district, B_u^{urb} are the befits of each urban center, and B_s^{eco} are the environmental benefits of each river reach. Equations (17) and (18) allocate water among uses and locations. Equation (17) ensures that water extractions at each demand node are lower than or equal to net water inflows in river reaches. Equation (18) indicate that basin water withdrawals cannot exceed water availability $\overline{W}$ in the basin.

The model is used to analyze the impact of droughts on the basin economic activities and environment. Water inflows into the system under normal weather conditions are 14,600 hm^3, corresponding to the average inflows in the 1980–2014 period [53]. Water inflows into the system are reduced by 40% under severe drought conditions. The

model is also used to analyze the economic and environmental effects of drought management policies. The drought policy scenarios considered are:

Institutional cooperation: Under drought conditions, the basin authority reduces water allocations for irrigation in proportion to drought intensity (fall in inflows). Consequently, the water shortfall is shared between irrigation districts, but it also reduces environmental flows. This is the policy currently applied in the Ebro basin.

Environmental institutional cooperation: Farmers receive the same allocations than under *institutional cooperation*, but then the basin authority purchases water from farmers for the environment in order to maximize social benefits, the sum of both private and environmental benefits. Water exchanges between irrigation districts are not allowed.

Water markets: Farmers receive the water allocations of institutional cooperation, but then these water allocations can be exchanged among irrigation districts, maximizing the private benefits of water use. There is no direct exchange of water between selling and buying irrigation districts, but rather the selling district reduces withdrawals and the buying district augments withdrawals in their respective river reaches.

Environmental water markets: Same water allocations than institutional cooperation. Water can be exchanged between irrigation districts, and also the basin authority participates in the water market by acquiring water to protect environmental benefits in river reaches. This policy enhances both private and environmental gains, so it is an appealing policy to capture the private benefits of markets while protecting ecosystems.

The baseline scenario assumes the policy currently applied under normal weather conditions. The minimum environmental streamflow at the mouth of the Ebro is set at 3,000 hm^3, and the urban water supply is guaranteed in all scenarios.

Results and discussion

In the *baseline scenario* with normal weather, the social benefits of water from economic activities and the environment are 3,442 M€, of which 629 M€ are from irrigation, 1,857 M€ from urban use, and 956 M€ from the environment. Water withdrawals are 5,380 Mm^3 in irrigation and 402 Mm^3 in urban use, while environmental flow at the mouth is 8,895 Mm^3. This environmental flow collects all water coming from stream flows across the basin, sustaining aquatic ecosystems (Table 2 and Table S1 in Supplementary Materials).

The irrigated area under normal weather is 529,000 ha, distributed between field crops (75%), fruit trees (20%) and vegetables (5%). Fruit trees and vegetables generate half of farmers' benefits. The cropping pattern is quite different by irrigation district, with Riegos del Alto Aragón and Bardenas specializing in field crops, Riegos del Jalón specializing in fruit trees, and Canal de Lodosa specializing in vegetables.

Institutional cooperation. Water availability is reduced 40% under drought conditions. In the *institutional cooperation* policy, water allocations to irrigation are a share of water inflows into the basin, and they are reduced in proportion to drought intensity. Water withdrawals for urban centers are maintained because of the priority of urban supply over other uses, including the environment. The decrease in the availability of water causes losses in irrigation and environmental benefits, reducing social benefits. During drought periods and under the *institutional cooperation* policy,

Table 2. Policies under drought conditions: institutional cooperation, environmental institutional cooperation, water markets, and environmental water markets.

Weather scenario	Normal	Drought			
Policy type	Baseline scenario	Institutional cooperation	Environmental institutional cooperation	Water markets	Environmental water markets
Water use (Mm^3)					
Water use	5,782	3,632	3,047	3,627	3,232
Irrigation	5,380	3,230	2,645	3,225	2,830
Urban	402	402	402	402	402
Water exchanges			585	235	780
Between irrigators				235	380
Between irrigators and environment			585		400
Environmental flow at mouth	8,895	5,350	5,540	5,345	5,435
Irrigation surface area (1,000 ha)					
Surface area	529	332	275	348	293
Field crops	400	219	165	229	182
Fruit trees	104	93	90	97	90
Vegetables	25	20	20	22	21
Private and environmental benefits (M€)					
Private benefits	2,486	2,321	2,332	2,340	2,346
Irrigation	629	464	475	483	489
Urban	1,857	1,857	1,857	1,857	1,857
Environmental benefits	956	761	834	719	826
Social benefits	3,442	3,082	3,105	3,059	3,118

irrigation withdrawals fall to 3,230 Mm^3 (-40%). This reduction leads to less irrigated area (-37%) and private benefits (-26%), compared to the *baseline scenario*. The streamflow at the river mouth drops from 8,895 Mm^3 to 5,350 Mm^3 under drought (Table 2).

Environmental benefits decrease by 20% during drought, affecting especially right bank tributaries of the Ebro, with diminished stream flows. The tributaries on the left bank provide most of the water in the Ebro, and pressures on environmental flows are mostly from irrigation withdrawals and, to a lesser extent, from variability in weather conditions. The effects of drought on the environment are more pronounced in the right bank of the Ebro, given the strong seasonality of flows on this bank. Water scarcity in

summer months determines the ecosystem sensitivity to drought. The ecosystems of the main stem of the Ebro have less environmental sensitivity than those of tributaries because the decline in flows is small (Fig 2 and S1 Fig). Under drought, urban benefits are maintained but irrigation and environmental benefits fall.

Fig 2. Environmental benefits of policies under normal and drought conditions

The *environmental institutional cooperation* policy reallocates water between irrigation and the environment to maximize social wellbeing. Social wellbeing is the sum of private and environmental benefits, minus the public expenses to buy irrigation water for the environment. Acquiring water for the environment improves ecosystem status, especially in areas with high potential for improvement, while maintaining irrigators' income. Under drought conditions the basin authority purchases 600 Mm^3 for €60 M. This reallocation increases environmental benefits by €70 M (9%) and irrigation benefits increase by nearly €9 M.

Water markets. Under the *water markets* policy, irrigation districts exchange water and maximize their private benefits, but environmental benefits are disregarded. Under drought, water withdrawals are 3,225 Mm^3 and cultivated area falls to 348,000 ha, with lower irrigation benefits. Flows at the river mouth are 5,345 Mm^3 under drought, well above the minimum environmental flow at 3,000 Mm^3. This minimum environmental flow is satisfied by all policy scenarios (Table 2, Fig 3). Irrigation districts exchange 235 Mm^3 under drought, where irrigation districts with low water efficiency and specialized in field crops sell water to irrigation districts with efficient irrigation systems and profitable crops. Water exchanges enable a larger cultivated area compared with the institutional cooperation policy. That is why crop water consumption (evapotranspiration) is higher under *water markets*, reducing basin stream flows and

environmental benefits. The *water markets* policy generates higher private benefits and lower environmental benefits compared with *institutional cooperation*.

Fig 3. Stream flows along the Ebro for each policy under drought conditions (Mm^3/year).

[Note below the figure] Minimum annual environmental flows, regulated by the Ebro Basin Authority, are: 300 Mm^3 at Mendavia under normal and drought conditions; 945 Mm^3 at Zaragoza under normal conditions and 640 Mm^3 under drought conditions; and 3,000 Mm^3 in Tortosa under normal and drought conditions [53].

Irrigation districts buying water expand withdrawals and deplete water streams in their river reach, while the opposite happening in selling districts. Consequently, water exchanges relieve environmental pressures in selling districts and aggravate pressures in buying districts. This can be observed in the Cinca watershed (Fig 2 in pink) where irrigation districts sell water and environmental benefits increase above any other policy, or in the Segre watershed (Fig 2 in green at right) where irrigation districts buy water and environmental benefits fall.

The purpose of the *environmental water markets* policy is to maximize social benefits by including both the private and public benefits of water, in order to internalize the external effects of water markets. The *environmental water markets* policy consists in water trading not only between irrigation districts but also with the environment, where the basin authority purchases water for the environment. Under drought conditions, water exchanges between irrigation districts are 380 Mm^3, and water exchanges between irrigation districts and the environment are 400 Mm^3. Irrigation withdrawals, irrigated area and crop production decrease compared with the *water markets* policy. The benefits of irrigation districts are obtained from crop production (393 M€) and from water trading with other districts (42 M€) and the environment (54 M€). The private benefits of irrigation under *environmental water*

markets are almost the same than under *water markets*, since crop production benefits and income from water sales are added in environmental water markets.

Environmental water markets and *environmental institutional cooperation* reduce water withdrawals, especially in river sections where environmental sensitivity is high. Water exchanges between irrigation and the environment increases environmental flows by reducing the cultivated area of field crops and fruit trees, while maintaining vegetables. The area of fruit trees decreases in irrigation districts withdrawing from river sections which are more environmentally sensitive, especially on the right bank.

Environmental institutional cooperation achieves a better ecosystem protection than *environmental water markets*, by acquiring almost 50% more water for the environment. Because there are no water exchanges among districts, farmers cannot take advantage of the private gains achieved under *environmental water markets*. Irrigation withdrawals under *environmental water markets* are around 200 Mm^3 larger than with *environmental institutional cooperation*, showing the trade-off between private and environmental benefits of these policies. *Environmental institutional cooperation* provides more environmental flows and better ecological status in all river reaches across the basin. In contrast, *environmental water markets* improve the ecological status in some river reaches at the expense of others reaches.

The spatial location of irrigation districts, the relationship between available water and water withdrawals, ecosystem sensitivity to water scarcity, crop patterns and irrigation technologies are the factors driving the impact caused by water scarcity on economic activities and the environment. Environmental and irrigation responses depend on spatial location, because water available and water withdrawal intensity are heterogeneous throughout the basin. Ecosystem sensitivity, the economic value of the ecosystem services, and alternative uses of water shape environmental flows. Under

drought, each policy results in different distribution of losses between private and environmental benefits and the ensuing trade-offs.

During droughts, *institutional cooperation* distributes losses to irrigation districts and the environment in proportion to water allocations in normal years. *Environmental institutional cooperation* enhances social benefits providing additional protection to the environment. *Water markets* maximize private benefits of irrigation but disregards environmental benefits, and *environmental water markets* deliver both private and environmental gains, capturing the private benefits of markets while protecting ecosystems. Water exchanges from irrigation to the environment enhances environmental benefits in the river reach of the exchange, and also in downstream river reaches, thus boosting the exchange benefits.

Reponses to drought entail maintaining high profitable crops and efficient irrigation systems, and therefore water scarcity has greater negative impacts on cultivation of less technically-advanced irrigation districts specialized in field crops. These irrigation districts with field crops and low water efficiency sell water to efficient and profitable irrigation districts and also to the environment.

The *water markets* policy provides the lowest social benefits because environmental losses overcome irrigation gains. The *environmental water markets* policy accounts for the market shortcomings by internalizing the negative external effects of economic activities, enhancing social welfare. However, the ecological status with this “environmental” trading improves in some river reaches but worsens in others. Equity considerations are better addressed by the *institutional cooperation* policy, which distributes proportionally the drought water scarcity among all irrigation districts and aquatic ecosystems in the basin. This policy not only protects the environment but also contributes to a more equitable distribution of water and the benefits it provides.

Environmental institutional cooperation further enhances social benefits by a full consideration of environmental benefits into water allocation decisions. The tradeoff between environmental and private benefits is obtained by comparing the outcomes of benefits under each policy. For example, there are gains in environmental benefits and losses in private benefits when *environmental institutional cooperation* is compared with *water markets*.

The range of the increase in environmental benefits between *environmental institutional cooperation* (high flow protection) and *water markets* (low flow protection) is quite close (115 M€ = 834-719, Table 1) to the environmental damages from water uses estimated by Garcia de Jalon et al. [69] in northern Spain. They estimate the environmental costs of streamflow variability induced by human extractions, applying the “polluter pays” principle. Their estimation of damages is 0.02 €/m^3, which in the case of the Ebro will amount to 107 M€ (0.02 €/m3□5,345□10^6 m^3). This similarity in the estimation of environmental damages in the two studies, that use quite different methodologies, strengthen the reliability of the damages found in this study.

Conclusions and policy implications

This paper presents the development and application of a hydroeconomic model that analyzes water allocation policies in the Ebro basin under water scarcity. The hydroeconomic approach has clear advantages in evaluating management and policy strategies for adaptation to climate change, by providing efficient water allocations across water uses and spatial locations. The contribution made by this study in comparison to previous studies is the inclusion of an environmental component linking stream flows with the status of aquatic ecosystems and their environmental benefits.

Most hydroeconomic models consider environmental protection by setting minimum flow requirements, rather than representing the complex ecological responses to stream flows that could be non-linear, dynamic and with regime shifts [23,70]. In this paper, the ecosystem status has been modeled and calibrated based on studies relating ecosystem habitat status with stream flows in the riverbeds of the Ebro basin. Then, the ecosystem status determines the environmental benefits provided to society.

The hydroeconomic model includes a regional economic component for irrigated agriculture and urban centers, and an environmental component. The purpose is to encompass both the spatial and sectoral interrelationships of water allocations in the basin. The model is used to analyze water allocation policies in the Ebro basin, a basin with several semi-arid regions where droughts trigger water scarcity problems and significant impacts on irrigation and the environment. This problem is common to arid and semi-arid basins worldwide, and it is expected to worsen as a consequence of climate change and pressures from economic and population growth. The methodology and results of this policy analysis could be applied to other basins facing similar problems of mounting withdrawals and ecosystems degradation.

Under drought conditions, the *water markets* policy generates higher private benefits than the current *institutional cooperation* policy, but attains lower environmental benefits. Irrigation districts gain from water trading, but environmental benefits are overlooked. The public good characteristics of environmental flows imply that ecosystem services are externalities not accounted by *water markets*. The market failure has to be corrected through mechanisms dealing with these negative externalities from excessive water withdrawals.

In the *environmental water markets* policy, the basin authority is a trading partner buying water for the river. The sharing of water between withdrawals for economic

sectors and environmental flows is based on the corresponding private and environmental benefits. Results from this “environmental” trading show that there are gains for both farmers and the environment, thus achieving higher social benefits. However, under this policy the ecosystem status improves in some river reaches but worsens in other river reaches. *Environmental water markets* require also the cooperation among stakeholders as an essential ingredient to curtail benefit losses from water scarcity, and achieve the “good ecological status” of water bodies promoted by European legislation [71–78]. Well functioning *environmental water markets* would enhance social benefits [75-78], but this entails the support of strong institutions fostering collective action [20].

The *environmental institutional cooperation* policy achieves a more equitably distribution of drought shortfalls among irrigation districts and attains the highest environmental protection. The institutional approach of the water authority is based on stakeholders’ cooperation and water management at basin level, and takes into account the interdependencies between sectors, spatial locations and the environment. This collaboration among water users, administrations and other stakeholders is essential for implementing sustainable water management.

The design and implementation of sustainable water management requires information on the response of ecosystems to water flows, and on the valuation of ecosystems services. Sustainable management alternatives have to be based on multidisciplinary research that requires considerable efforts in terms of resources and time, and this research is lacking at present in most basins around the world. This information is needed to gain the support of stakeholders in reversing the current global degradation of water resources.

The principle of water management at basin level enshrined in Spanish water authorities and the involvement of stakeholders in decision making facilitates policy interventions adapted to spatial locations. This is important because the spatial heterogeneity of economic and environmental effects of droughts implies that scarcity problems have to be addressed with local actions in river sections. This is the approach taken by the water authority, where watershed boards manage water withdrawals by adjusting them to local conditions.

The challenge for water management is balancing the effects of water allocations between economic activities and environmental flows in decision-making. Market policies do not internalize the externalities generated by private water usage, and therefore interventions are needed to deal with market failures from an institutional cooperation perspective. The situation in Spanish basins shows that basin stream flows have gradually decreased in recent decades. Consequently, the decline in environmental flows is damaging aquatic ecosystems and their environmental services. Private benefits could increase in the short run at the expense of public environmental benefits, but in the long run the degradation of hydrological systems would become unsustainable, with strong negative impacts on both economic activities and ecosystems.

Acknowledgements

This study has been financed by the projects INIA RTA2014-00050-00-00 and INIA RTA2017-00082-00-00 of the Ministry for Science and Innovation, partly financed by European ERDF funds, and by support received from the ECONATURA research group of the Government of Aragon. Daniel Crespo has conducted this study with the support of a PhD grant from INIA. Part of the work by Daniel Crespo was developed at the School of Public Policy at UC Riverside. Also, Daniel Crespo would like to acknowledge Dr. Darrel Jenerette (UCR), Dr. Louis Santiago (UCR), Dr. Kurt E. Anderson (UCR) and Dr. Sergio Rey (UCR) for their advices and recommendations

about ecosystem and spatial modeling. Ariel Dinar would like to acknowledge support from the W4190 Multistate NIFA-USDA-funded Project, “Management and Policy Challenges in a Water-Scarce World”. Special assistance has been provided by María Ángeles Lorenzo and Daniel Isidoro (CITA-DGA), and by Rogelio Galván and Miguel Ángel García Vera (CHE-MITECO).

References

1. Millennium Ecosystem Assessment (Program) (Eds.) (MEA). Ecosystems and human well-being. Washington, DC.: Island Press; 2005.

2. Arthington AH , ebrary, Inc.,. Environmental flows saving rivers in the third millennium. Berkeley: University of California Press; 2012.

3. Dasgupta Partha Great Britain, Treasury,. Economics of Biodiversity : The Dasgupta Review. 2021.

4. Booker JF Young, Robert A, Colorado Water Resources Research Institute,. Economic impacts of alternative water allocation institutions in the Colorado River basin. Fort Collins, Colo.: Colorado Water Resources Research Institute, Colorado State University; 1991.

5. Booker JF, Young RA. Modeling Intrastate and Interstate Markets for Colorado River Water Resources. J Environ Econ Manag. 1994;26: 66–87. doi:10.1006/jeem.1994.1005

6. Brown T, Harding B, Payton E. Marginal economic value of streamflow: A case study for the Colorado River Basin. Water Resour Res. 1990;26: 2845–2859. doi:10.1029/WR026i012p02845

7. Cai X, McKinney DC, Lasdon LS. A framework for sustainability analysis in water resources management and application to the Syr Darya Basin: Sutainability analysis in water resources management. Water Resour Res. 2002;38: 21-1-21–14. doi:10.1029/2001WR000214

8. Divakar L, Babel MS, Perret SR, Gupta AD. Optimal allocation of bulk water supplies to competing use sectors based on economic criterion – An application to the Chao Phraya River Basin, Thailand. J Hydrol. 2011;401: 22–35. doi:10.1016/j.jhydrol.2011.02.003

9. Ringler C, von Braun J, Rosegrant M. Water policy analysis for the Mekong River Basin. Water Int. 2004;29: 30–42. doi:10.1080/02508060408691746

10. Babel MS, Gupta AD, Nayak DK. A Model for Optimal Allocation of Water to Competing Demands. Water Resour Manag. 2005;19: 693–712. doi:10.1007/s11269-005-3282-4

11. Booker JF, Michelsen AM, Ward FA. Economic impact of alternative policy responses to prolonged and severe drought in the Rio Grande Basin: Policy response to drought. Water Resour Res. 2005;41: 1–15. doi:10.1029/2004WR003486

12. Ringler C, Cai X. Valuing Fisheries and Wetlands Using Integrated Economic-Hydrologic Modeling—Mekong River Basin. J Water Resour Plan Manag. 2006;132: 480–487. doi:10.1061/(ASCE)0733-9496(2006)132:6(480)

13. Ward FA, Pulido-Velázquez M. Efficiency, equity, and sustainability in a water quantity–quality optimization model in the Rio Grande basin. Ecol Econ. 2008;66: 23–37. doi:10.1016/j.ecolecon.2007.08.018

14. Ward FA, Pulido-Velazquez M. Economic Costs of Sustaining Water Supplies: Findings from the Rio Grande. Water Resour Manag. 2012;26: 2883–2909. doi:10.1007/s11269-012-0055-8

15. Ward FA, Lynch TP. Is dominant use management compatible with basin-wide economic efficiency? Water Resour Res. 1997;33: 1165–1170. doi:10.1029/97WR00273

16. Grafton RQ, Chu HL, Stewardson M, Kompas T. Optimal dynamic water allocation: Irrigation extractions and environmental tradeoffs in the Murray River, Australia. Water Resour Res. 2011;47. doi:10.1029/2010WR009786

17. Grossmann M, Dietrich O. Integrated Economic-Hydrologic Assessment of Water Management Options for Regulated Wetlands Under Conditions of Climate Change: A Case Study from the Spreewald (Germany). Water Resour Manag. 2012;26: 2081–2108. doi:10.1007/s11269-012-0005-5

18. Bekchanov M, Ringler C, Bhaduri A, Jeuland M. Optimizing irrigation efficiency improvements in the Aral Sea Basin. Water Resour Econ. 2016;13: 30–45. doi:10.1016/j.wre.2015.08.003

19. Bekchanov M, Ringler C, Bhaduri A, Jeuland M. How would the Rogun Dam affect water and energy scarcity in Central Asia? Water Int. 2015;40: 856–876. doi:10.1080/02508060.2015.1051788

20. Bekchanov M, Bhaduri A, Ringler C. Potential gains from water rights trading in the Aral Sea Basin. Agric Water Manag. 2015;152: 41–56. doi:10.1016/j.agwat.2014.12.011

21. Yang Y-CE, Cai X. Reservoir Reoperation for Fish Ecosystem Restoration Using Daily Inflows—Case Study of Lake Shelbyville. J Water Resour Plan Manag. 2011;137: 470–480. doi:10.1061/(ASCE)WR.1943-5452.0000139

22. Bryan BA, Higgins A, Overton IC, Holland K, Lester RE, King D, et al. Ecohydrological and socioeconomic integration for the operational management of environmental flows. Ecol Appl. 2013;23: 999–1016. doi:10.1890/12-2104.1

23. Momblanch A, Connor JD, Crossman ND, Paredes-Arquiola J, Andreu J. Using ecosystem services to represent the environment in hydro-economic models. J Hydrol. 2016;538: 293–303. doi:10.1016/j.jhydrol.2016.04.019

24. Ahmadi A, Karamouz M, Moridi A, Han D. Integrated Planning of Land Use and Water Allocation on a Watershed Scale Considering Social and Water Quality Issues. J Water Resour Plan Manag-ASCE. 2012;138: 671–681. doi:10.1061/(ASCE)WR.1943-5452.0000212

25. Connor JD, Franklin B, Loch A, Kirby M, Wheeler SA. Trading water to improve environmental flow outcomes: Environmental Water Trade. Water Resour Res. 2013;49: 4265–4276. doi:10.1002/wrcr.20323

26. Kahil MT, Dinar A, Albiac J. Modeling water scarcity and droughts for policy adaptation to climate change in arid and semiarid regions. J Hydrol. 2015;522: 95–109. doi:10.1016/j.jhydrol.2014.12.042

27. Potschin MB, Haines-Young RH. Ecosystem services: Exploring a geographical perspective. Prog Phys Geogr. 2011;35: 575–594. doi:10.1177/0309133311423172

28. Horne A. An approach to efficiently managing environmental water allocations. University of Melbourne, Department of Civil and Environmental Engineering. 2009.

29. Poff NL, Zimmerman JKH. Ecological responses to altered flow regimes: a literature review to inform the science and management of environmental flows: Review of altered flow regimes. Freshw Biol. 2010;55: 194–205. doi:10.1111/j.1365-2427.2009.02272.x

30. Andersson E, Nilsson C, Johansson ME. Effects of river fragmentation on plant dispersal and riparian flora. Regul Rivers Res Manag. 2000;16: 83–89. doi:10.1002/(SICI)1099-1646(200001/02)16:1<83::AID-RRR567>3.0.CO;2-T

31. Anderson EP, Freeman MC, Pringle CM. Ecological consequences of hydropower development in Central America: impacts of small dams and water diversion on neotropical stream fish assemblages. River Res Appl. 2006;22: 397–411. doi:10.1002/rra.899

32. Humphries P, Lake PS. Fish larvae and the management of regulated rivers. 2000; 12.

33. Acreman MC, Dunbar MJ. Defining environmental river flow requirements? A review. Hydrol Earth Syst Sci Discuss. 2004;8: 861–876.

34. Arthington AH, Bunn SE, Poff NL, Naiman RJ. The challange of providing environmental flow rules to sustain river ecosystems. Ecol Appl. 2006;16: 1311–1318. doi:10.1890/1051-0761(2006)016[1311:TCOPEF]2.0.CO;2

35. Jørgensen S, editor. Handbook of Ecological Indicators for Assessment of Ecosystem Health. CRC Press; 2005. doi:10.1201/9780203490181

36. Rolls RJ, Heino J, Ryder DS, Chessman BC, Growns IO, Thompson RM, et al. Scaling biodiversity responses to hydrological regimes. Biol Rev. 2018;93: 971–995. doi:10.1111/brv.12381

37. Davey AJH, Kelly DJ. Fish community responses to drying disturbances in an intermittent stream: a landscape perspective. Freshw Biol. 2007;52: 1719–1733. doi:10.1111/j.1365-2427.2007.01800.x

38. de Macedo-Soares PHM, Petry AC, Farjalla VF, Caramaschi EP. Hydrological connectivity in coastal inland systems: lessons from a Neotropical fish metacommunity. Ecol Freshw Fish. 2010;19: 7–18. doi:10.1111/j.1600-0633.2009.00384.x

39. Laske SM, Haynes TB, Rosenberger AE, Koch JC, Wipfli MS, Whitman M, et al. Surface water connectivity drives richness and composition of Arctic lake fish assemblages. Freshw Biol. 2016;61: 1090–1104. doi:10.1111/fwb.12769

40. Lamouroux N, Poff N, Angermeier P. Intercontinental convergence of stream fish community traits along geomorphic and hydraulic gradients. ECOLOGY. 2002;83: 1792–1807. doi:10.1890/0012-9658(2002)083[1792:ICOSFC]2.0.CO;2

41. Poff NL, Richter BD, Arthington AH, Bunn SE, Naiman RJ, Kendy E, et al. The ecological limits of hydrologic alteration (ELOHA): a new framework for developing regional environmental flow standards: Ecological limits of hydrologic alteration. Freshw Biol. 2010;55: 147–170. doi:10.1111/j.1365-2427.2009.02204.x

42. Potschin-Young M. Intermediate ecosystem services: An empty concept? Ecosyst Serv. 2017; 4.

43. Boyd J, Banzhaf S. What are ecosystem services? The need for standardized environmental accounting units. Ecol Econ. 2007;63: 616–626. doi:10.1016/j.ecolecon.2007.01.002

44. TEEB. The Economics of Ecosystems and Biodiversity: Mainstreaming the Economics of Nature: A Synthesis of the Approach, Conclusions and Recommendations of TEEB. London and Washington: Ecological and Economic Fundation, Earthscan; 2010.

45. Costanza R, d'Arge R, de Groot R, Farber S, Grasso M, Hannon B, et al. The value of the world's ecosystem services and natural capital (Reprinted from Nature, vol 387, pg 253, 1997). Ecol Econ. 1998;25: 3–15. doi:10.1016/S0921-8009(98)00020-2

46. Haines-Young R, Potschin M. Common International Classification of Ecosystem Services (CICES) V5.1 and Guidance on the Application of the Revised Structure. Nottingham: European Environmental Agency; 2019 p. 53.

47. Costanza R. Valuing natural capital and ecosystem services toward the goals of efficiency, fairness, and sustainability. Ecosyst Serv. 2020;43: 101096. doi:10.1016/j.ecoser.2020.101096

48. Tharme R. A global perspective on environmental flow assessment: Emerging trends in the development and application of environmental flow methodologies for rivers. River Res Appl. 2003;19: 397–441. doi:10.1002/rra.736

49. Sale MJ, Brill ED, Herricks EE. An approach to optimizing reservoir operation for downstream aquatic resources. Water Resour Res. 1982;18: 705–712. doi:10.1029/WR018i004p00705

50. Cardwell H Jager, Henriette I, Sale, Michad J,. Designing Instream Flows to Satisfy Fish and Human Water Needs. J Water Resour Plan Manag. 1996;122: 356.

51. Lamouroux N, Jowett IG. Generalized instream habitat models. Can J Fish Aquat Sci. 2005;62: 7–14. doi:10.1139/f04-163

52. Wilding TK, Bledsoe B, Poff NL, Sanderson J. Predicting habitat response to flow using generalized habitat models for trout in Rocky Mountain streams. River Res Appl. 2014;30: 805–824. doi:10.1002/rra.2678

53. Confederación Hidrográfica del Ebro (CHE). Plan Hidrológico de la Demarcación Hidrográfica del Ebro, Memoria CHE. Ministerio de Agricultura y Medio Ambiente (MAGRAMA); 2015.

54. Crespo D, Albiac J, Kahil T, Esteban E, Baccour S. Tradeoffs between Water Uses and Environmental Flows: A Hydroeconomic Analysis in the Ebro Basin. Water Resour Manag. 2019;33: 2301–2317. doi:10.1007/s11269-019-02254-3

55. Ministerio de Medio Ambiente y Medio Rural y Marino (MARM). Valoración de los activos naturales de España. Madrid: Ministerio de Medio Ambiente y Medio Rural y Marino, Centro de Publicaciones; 2010.

56. Howitt RE. Positive Mathematical Programming. Am J Agric Econ. 1995;77: 329. doi:10.2307/1243543

57. Dagnino M, Ward FA. Economics of Agricultural Water Conservation: Empirical Analysis and Policy Implications. Int J Water Resour Dev. 2012;28: 577–600. doi:10.1080/07900627.2012.665801

58. Ministerio de Medio Ambiente, Rural y Marino (MARM). Análisis de la economía de los sistemas de producción: Resultados técnico-económicos de explotaciones agrícolas de

Aragón en 2009. Madrid: Subsecretaria de Medio Ambiente, Rural y Marino, MARM; 2010.

59. Ministerio de Agricultura y Medio Ambiente (MAGRAMA). Anuario de Estadísticas Agrarias. Madrid: Ministerio de Agricultura y Medio Ambiente (MAGRAMA); 2015.

60. Instituto Nacional de Estadística (INE). Censo Agrario 2009. Madrid: INE; 2009.

61. Gobierno de Aragón (DGA). Base de datos 1T de superficies de cultivos por término municipal para El Gobierno de Aragón 2009. Zaragoza: Departamento de Desarrollo Rural y Sostenibilidad; 2009.

62. Generalitat de Cataluya (GC). Superfícies Municipals Dels Conreus Agrícoles. Any 2009. Barcelona: Departament d’Agricultura, Ramaderia, Pesca, Alimentació i Medi Natural; 2009.

63. Gobierno de Navarra (GN). Base de datos 1T de superficies de cultivos por término municipal para el Gobierno de Navarra 2009. Departamento de Desarrollo Rural, Medio Ambiente y Administración Local.; 2009.

64. Arbués F, Barberán R, Villanúa I. Price impact on urban residential water demand: A dynamic panel data approach. Water Resour Res. 2004;40. doi:10.1029/2004WR003092

65. Arbués F, García-Valiñas MÁ, Villanúa I. Urban Water Demand for Service and Industrial Use: The Case of Zaragoza. Water Resour Manag. 2010;24: 4033–4048. doi:10.1007/s11269-010-9645-5

66. Troy A, Wilson MA. Mapping ecosystem services: Practical challenges and opportunities in linking GIS and value transfer. Ecol Econ. 2006;60: 435–449. doi:10.1016/j.ecolecon.2006.04.007

67. Troy A, Bagstad K. Estimating Ecosystem Services in Southern Ontario. Spatial Informatics Group, LLC 3248 Northampton Ct. Pleasanton, CA 94588 USA; 2009.

68. Andreadis KM, Schumann GJ-P, Pavelsky T. A simple global river bankfull width and depth database: Data and Analysis Note. Water Resour Res. 2013;49: 7164–7168. doi:10.1002/wrcr.20440

69. García de Jalón S, González del Tánago M, Alonso C, García de Jalón D. The Environmental Costs of Water Flow Regulation: an Innovative Approach Based on the ‘Polluter Pays’ Principle. Water Resour Manag. 2017;31: 2809–2822. doi:10.1007/s11269-017-1663-0.

70. Bekchanov M, Sood A, Pinto A, Jeuland M. Systematic Review of Water-Economy Modeling Applications. J Water Resour Plan Manag. 2017;143: 04017037. doi:10.1061/(ASCE)WR.1943-5452.0000793

71. Esteban E, Albiac J. Groundwater and ecosystems damages: Questioning the Gisser–Sánchez effect. Ecol Econ. 2011;70: 2062–2069. doi:10.1016/j.ecolecon.2011.06.004

72. Esteban E, Dinar A. Modeling sustainable groundwater management: Packaging and sequencing of policy interventions. J Environ Manage. 2013;119: 93–102. doi:10.1016/j.jenvman.2012.12.047

73. Kahil MT, Dinar A, Albiac J. Cooperative water management and ecosystem protection under scarcity and drought in arid and semiarid regions. Water Resour Econ. 2016;13: 60–74. doi:10.1016/j.wre.2015.10.001

74. Giuliani M, Castelletti A. Assessing the value of cooperation and information exchange in large water resources systems by agent-based optimization: MAS Framework for Large Water Resources Systems. Water Resour Res. 2013;49: 3912–3926. doi:10.1002/wrcr.20287

75. Ancev T. The role of the commonwealth environmental water holder in annual water allocation markets. Aust J Agric Resour Econ. 2015;59: 133–153. doi:10.1111/1467-8489.12054

76. Howitt RE, Medellín-Azuara J, MacEwan D, Lund JR. Calibrating disaggregate economic models of agricultural production and water management. Environ Model Softw. 2012;38: 244–258. doi:10.1016/j.envsoft.2012.06.013

77. Grafton RQ, Libecap G, McGlennon S, Landry C, O'Brien B. An Integrated Assessment of Water Markets: A Cross-Country Comparison. Rev Environ Econ Policy. 2011;5: 219–239. doi:10.1093/reep/rer002

78. Horne AC, O'Donnell EL, Loch AJ, Adamson DC, Hart B, Freebairn J. Environmental water efficiency: Maximizing benefits and minimizing costs of environmental water use and management. Wiley Interdiscip Rev Water. 2018;5: e1285. doi:10.1002/wat2.1285

Figure1

Click here to access/download;Figure;Fig1.tif

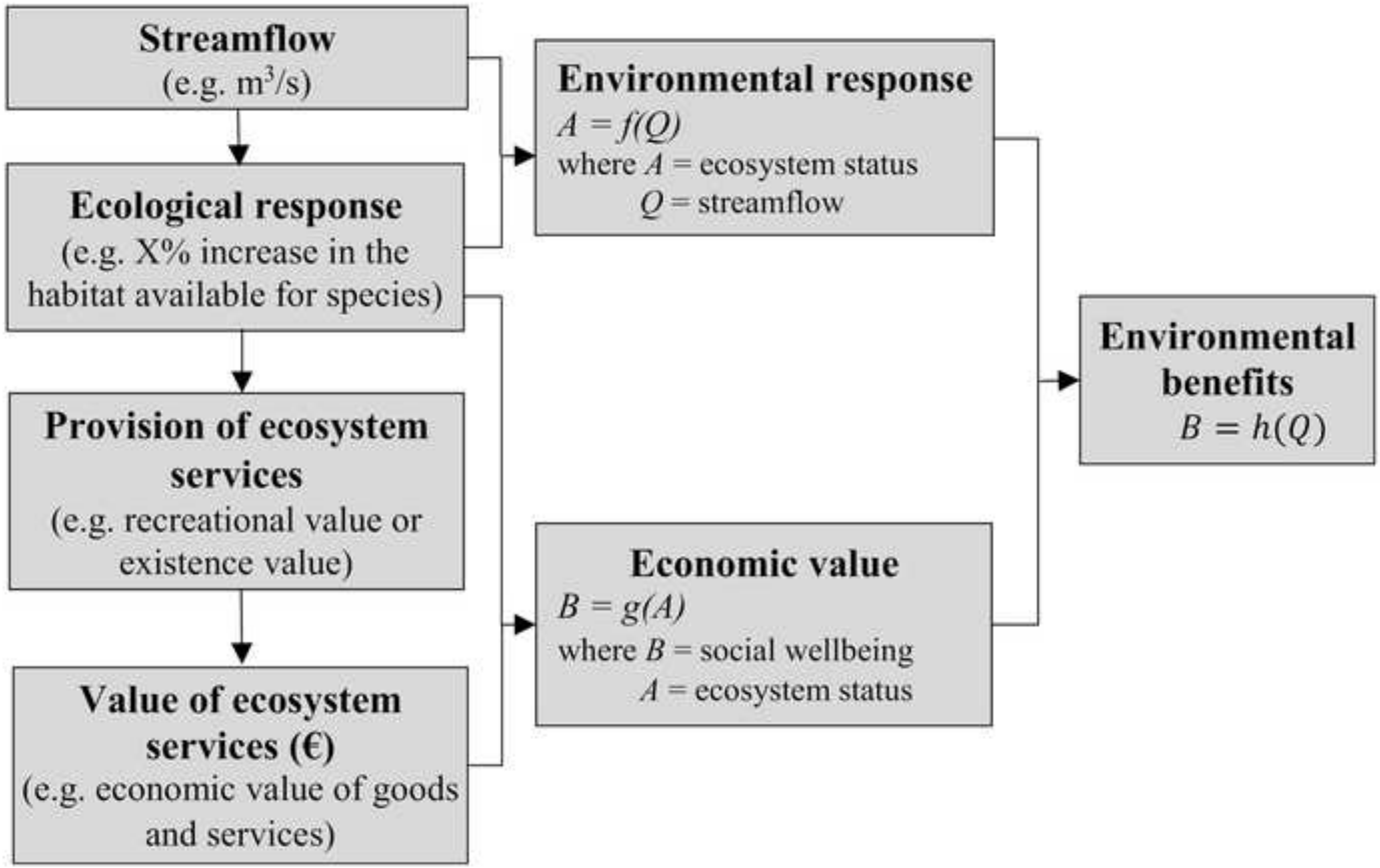

Figure2 Click here to access/download;Figure;Fig2.tif

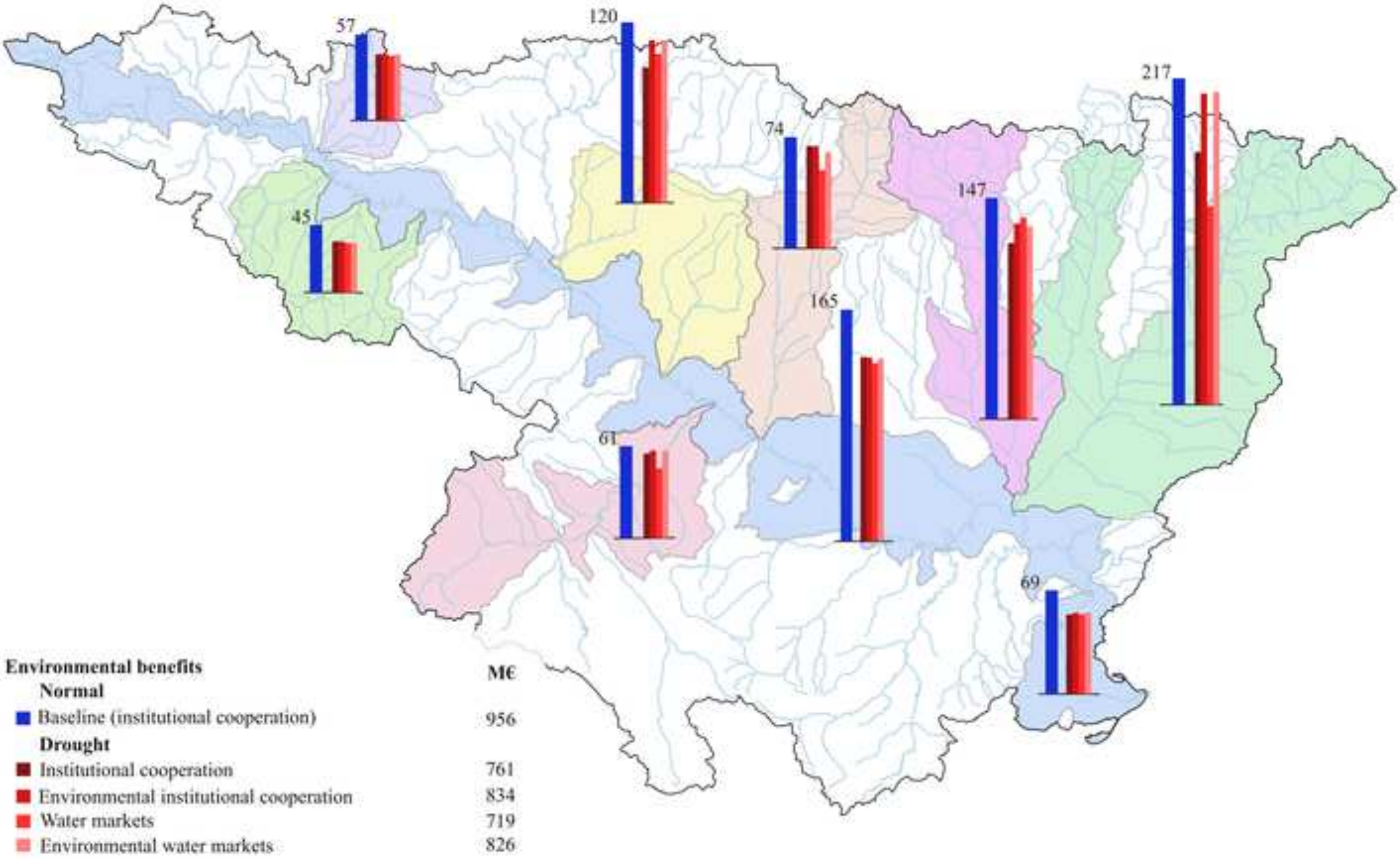

Figure3

Click here to access/download;Figure;Fig3.tif

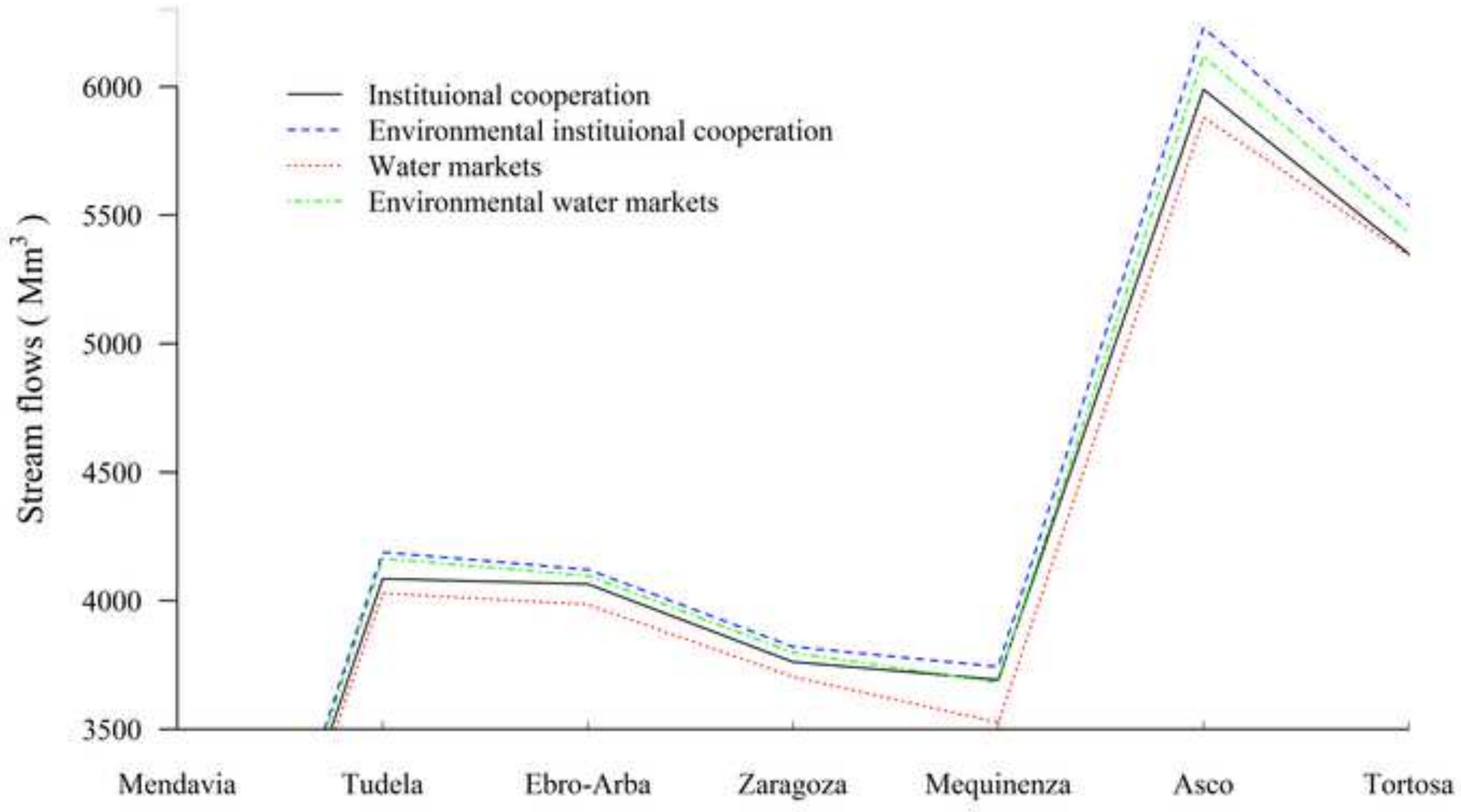

Click here to access/download;Figure;S1_Fig.tif

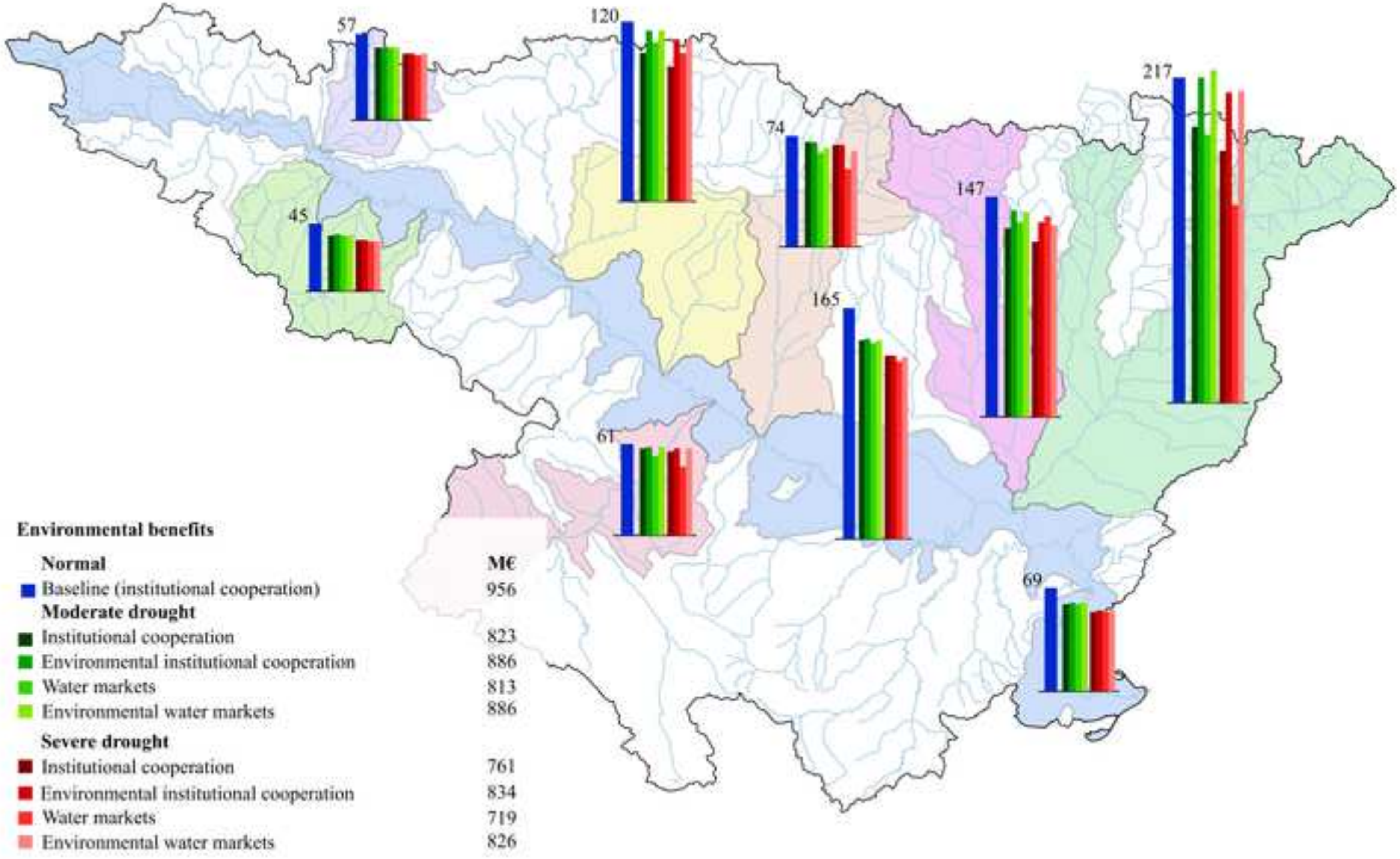

Click here to access/download
Supporting Information
SuplementaryMaterials.docx
