# Supplementary material for: Integrating ecosystem benefits for sustainable water allocation in hydroeconomic modeling
Source: PLoS One. 2022 May 5;17(5):e0267439. doi: 10.1371/journal.pone.0267439 (PMC9070880; doi:10.1371/journal.pone.0267439)
Supplement: Attachment — Submitted filename: Response to Reviewers.docx [file pone.0267439.s006.docx]

Response Letter

**Editor**

Thank you for the opportunity to revise and resubmit our manuscript. The data underlying the findings are now being provided. They are included in the GAMS code of the model, which is made available in Supplementary Materials.

Below are our responses in red to each of the reviewer comments.

**Reviewer 1**
Comments
This paper innovates on the existing literature by integrating ecosystems benefits calculations into a hydro-economic optimization model, which is a useful contribution to the literature. It should also be noted that the authors show an in-depth knowledge of the literature and relevant methods.

Thank you for your overall assessment of the contribution of our work. In particular, we are very pleased that you found our paper interesting and aligned with the aims and scope of PLOS ONE. The feedback/recommendations you provided have greatly helped us improve different aspects of our paper. We have reflected upon your recommendations. We have also provided point-by-point answers to the queries below with hope that our responses meet your expectations, and the paper is now clearer about the methodology and the results.

However, the paper contains too much general content on the literature and methods while not elaborating in enough detail the specifics of their own research. The introduction and methods sections contain too much general discussion of the literature and too many vague assertions. For example, paragraphs 2-4 in the introduction contain neither a) any citations nor b) any concrete examples. The paper would be improved by the judicious use of real-world examples/case studies to motivate the topic.

We have modified the introduction and method sections to specify the type of discussions in the literature and avoid vague assertions by providing citations and concrete examples for a better motivation (new lines in introduction: 44-47, 49-53, 57-61, and citations)

Relatedly, the Methods section should focus almost entirely on authors’ methodology rather than addressing the literature and the discussion of the Ebro basin. The discussion of the Ebro basin should be condensed and it belongs in its own section, which could include discussion of the data used. The Methods section could be dramatically reduced; I think that none, or almost none, of the content before the Environmental response function sub-section belongs in the Methods. Given the centrality of the WUA function to the paper, I think it merits a little more elaboration than given in lines 266-269. A diagram to aid reader intuition would be helpful.

We added a new section on the Ebro basin, placed before the Methods section, describing the basin, the institutional arrangements, and the setting of environmental flows. The section has been condensed and includes also the sources of the data used (new lines 151-195).

The Methods section has been significantly reduced. The text before the Environment response function sub-section (old lines 145-217) has been strongly condensed (new lines 197-221).

The explanation of the WUA function has been expanded, and the text covers now the new lines 223-269. A new diagram (new Figure 1) has been elaborated to aid reader intuition.

A few sentences to explain the importance of the Ebro basin would be useful. While statistics on the basin are offered, there is little or no context offered as to the importance of the basin relative to Spanish agriculture or the EU as a whole. A map of Spain or perhaps the Iberian Peninsula with the Ebro basin called out would be useful if space allows.

We explain now the importance of the Ebro basin, giving context on the large extent of the basin and the size of the water based economic activities such as irrigation and hydropower (new lines 152-154). A map of the location of the Ebro basin in Spain is now included in Figure 3 (old Figure 2).

The exposition of the mathematical programming model needs to be re-worked. There are a number of issues:
1. I’m not sure exactly what you mean by a “reduced-form” hydrological model in this context.

We indicate now in the text (new lines 276-279) that “reduced form” means a simplified hydrology.

Developing the hydrologic part of the model is a time consuming and complex task that involves detailed hydrologic knowledge and highly-disaggregated biophysical information that may not be available, requiring advanced modeling abilities that could represent the complex hydrological relationships. Moreover, hydrologic and economic models usually have different resolution techniques, and spatial and temporal scales, which further complicate their linkage. An alternative approach is to use aggregated historical data provided by water authorities, together with simulated data and network topology from existing hydrologic models. This method is a quick and credible way to build a reduced form hydrological model of the studied river basin. A full-fledged hydrology model is available for the Ebro developed with the AQUATOOL model used by the Ebro Water Authority, which includes all stream flows (and groundwater bodies) in the basin. But using this very detailed hydrological network of the basin is beyond the scope of our study, because of the complexity of coupling it with the economic activities in the basin. We now explain in the new lines 276-279 what the “reduced form” means, including a citation of Cai et al. (2003) [52bis] on the issue.

Equations 1-3 give a system of equations that encapsulate the hydrology. To me a reduced-form model would collapse everything into a single equation. Not a major issue but worth considering if this is the best description.

The three equations (now in new lines 284-286) represent the mass balance in river reach d, the flow continuity between river reach d+1 and river reach d, and the minimum environmental flow in reach d.

The key vector equation to implement this hydrological information on mass balance and continuity in the code of the GAMS optimization package takes the following form:

Rivers_e (river).. X_v(river) =E= sum(inflow, Bv_p(inflow, river) * X_v(inflow)) +

sum(riverp, Bv_p(riverp, river) * X_v(riverp)) +

sum(divert, Bv_p(divert, river) * X_v(divert)) +

sum(returnc, Bv_p(returnc, river) * X_v(returnc))+

sum(returnp, Bv_p(returnp, river) * X_v(returnp))+

sum(unmeasure, Bv_p(unmeasure, river) * X_v(unmeasure));

where X_v(river) is the streamflow in each river gage measurement node, inflow are the headwater flow nodes, riverp are river gage measurement nodes, divert are the diversion nodes, returnc are return flows at canal level nodes, returnp are return flows at parcel level nodes and at urban level nodes, and unmeasured are unmeasured use flows nodes.

The matrix Bv_p is the hydrologic balance map linking the streamgages (river) [in columns] with the sources or uses of water (inflow, river, divert, returnc, returnp, unmeasured) [in rows], with elements 1 (such as headwaters) and -1 (such as diversions).

The following figure shows the network of nodes, stream flows and canals in the basin. This figure is now included in the text (new Figure 2)

You can check the details of the hydrological component directly in GAMS, because we are including now the GAMS code of the model in the Supplementary Materials. We indicate now in the text (new lines 295-297) that the details on the hydrological component can be found in the Supplementary Materials (GAMS code).

All this information is synthetized in equations 1-3, but cannot be collapsed into a single one dimension equation. As indicated above we now explain in the text the meaning of “reduced-form” hydrological component.

2. The urban sector sub-model is problematic. How can you claim to maximize PS + CS given that prices are exogenous? That is, if you fix prices you know the equilibrium quantity. Moreover, as the optimization problem is stated, Q_s = Q_d is pre-determined. This all just collapses into a constraint requiring that urban demand must be met. The results further emphasize this point; urban water use and benefits are constant across all scenarios. I strongly suggest that the authors consider reducing equations 10-12 to a single constraint and/or restating exactly what the model is doing.

You are right, we are not maximizing the consumer and producer surpluses because of the institutional setting in Spain that gives absolute priority to urban use over any other water use. Also, in the case of the Ebro urban use (630 Mm3) is a small share of total water withdrawals (8460 Mm3), so urban withdrawals would be maintained even under very severe droughts. In case of basins with large shares of urban use and severe drought, urban use (and benefits) would be reduced, even with urban priority.

The reason we maintain equations 10-12 is to explain how we obtain urban benefits, which are the main component of the private and social benefits (75%, 54% respectively). We think that maintaining the equations can be useful for running the model in other basins or countries, where there is not priority of urban use, or where there are water markets allowing trading between the urban and irrigation sectors.

3. Sector sub-models are detailed as standalone models and then later combined into one integrated model. Are any of the models run in isolation, e.g., to calibrate one sector before being incorporated in the larger model? As currently written, the use of objective functions is confusing. That is, each sector has an objective function and then you refer to equations 1-15 in the larger model. But that model has its own over-arching objective function. So, I would probably change equation 4, for example, to read something like B = Sum(C X), removing the Max function.

You are correct, the three sector sub-models are described as standalone, and later combined into an integrated model. But the sub-models are not run in isolation from each other.

Calibration of the hydrological component is conducted using the full integrated model under baseline conditions, as explained in new lines 299-301. Calibration of the agricultural component is conducted using a variant of PMP developed by Dagnino and Ward (2012), in which parameters are estimated for a linear crop yield function (Eq. 9 in the text) based on the first-order conditions for profit maximization. This PMP procedure is embedded in the GAMS code provided in the Supplementary Materials.

Following your suggestion, Eq. 4 has been changed (new line 314), and now we indicate in the text (new lines 311-313) that irrigation benefits are part of the objective function of the integrated model, which is maximized. Eq. 10 for urban benefits has also been changed (new line 351).

4. The geography of the sectors and how they fit together is not at all clear. For example, how do cities (u) relate to ecosystem areas (s)? Figures 2-4 don’t seem to clarify this point. A network diagram would be very useful here.

A network diagram has been added (Figure 2) showing the location of the network of nodes, stream flows and canals in the basin. Environmental benefits depend on the stream flow levels in the respective river reaches, so the impacts on environmental benefits in river reaches are mostly driven by the withdrawals from irrigation districts affecting downstream river reaches, which are much bigger than urban withdrawals. However, urban withdrawals will also affect downstream river reaches and their dependent ecosystems.

5. How many observations were used to fit the regressions in Table 1? This may be indicated elsewhere in the text but the reader should not have to hunt them down. This info should be in the table or the caption.

We indicate now in Table 1 the number of observations used to fit each regression.

6. I’m confused by the 3 levels of ecosystem values discussed in lines 438-447. The equations above this point explain how you calculate the environmental benefits. Given that this is endogenous to the model, why do you then bring up the 3 levels of values? It sounds like you’re overriding the values from the model. Can you explain this better?

Right. Environmental benefits are endogenous to the model. For each river reach, these benefits $B_{s}^{eco}$ depend on the ecosystem health status $EW_{s}^{eco}$ (driven by streamflow and taking values from zero to one), the length $l_{s}$ of the reach (km), and the economic value of ecosystem services $VE_{s}$ (€/km), as stated in equation 15. The ecosystem health status for each river reach is calculated endogenously by the model, and then this is multiplied by the length of the reach, and by the economic valuation of ecosystems taken from published studies in the literature (these values are exogenous to our model). Since ecosystem values in basins are spatially heterogeneous, we distinguish (from the literature) a range, and use three values for $VE_{s}$ which change by location. As indicated in the text, we consider a low value (0.072 M€/km) for river sections with moderate environmental value in the main steam of the Ebro and in some right bank tributaries, a medium value (0.180 M€/km) for non-mountain Ebro tributaries, and a high value (0.450 M€/km) for mountain river reaches and also for the Ebro Delta.

We now explain in new lines 399-414, how the values of $VE_{s}$ are obtained.

7. Why do you not have a mathematical description of the institutional scenarios? That is, you should be able to modify the model by using an alternate version of the relevant equations or constraints. You may need to add additional equations/constraints that are scenario specific. By not clarifying in this manner, you leave it open to the reader’s interpretation how you implement the scenarios and whether it is a logical way of doing so.

There are four policies to deal with drought-induced water scarcity. The scenarios are the combination of the drought situation with the four policies to address drought, which are the following:

- Institutional cooperation, where the basin authority (with irrigation district representatives inside taking decisions and enforcing, therefore cooperating) reduces water allocations to irrigation districts in proportion to the fall of inflows from drought.

This scenario is implemented in the model by reducing the baseline allowed withdrawals (normal weather) of irrigation districts in proportion to the water shortfall of drought, which is the current policy in the basin.

- Water markets, where water allocations to irrigation districts are also reduced in proportion to shortfalls, but then irrigation districts can trade water between districts. This is implemented in the model by introducing a restriction that limits the sum of reduced water allocations to districts, instead of limiting the (reduced) water allocation to each district, and maximizing irrigation benefits which results in water trading among districts.

- Environmental institutional cooperation, where environmental benefits are included in the objective function of the integrated model. As in the Institutional cooperation policy, the water allocation to irrigation district is reduced in proportion to the shortfall, but then the Water Authority can buy water from irrigation districts to achieve the optimal solution found for stream flows in rivers when the environmental benefits are included in the model objective function.

- Environmental water markets, where environmental benefits are included in the objective function of the model. The irrigation districts trade the reduced water allocation among themselves, and the Water Authority can buy water from irrigation districts to achieve the optimal solution found for stream flows resulting from the inclusion of environmental benefits in the objective function.

We include now in the sub-section “Modeling policy scenarios” the changes in the mathematical description corresponding to each policy scenario (new lines 445-447, 452-454, 460-462, 468-470).

8. The distinction between the baseline and the Institutional Cooperation scenarios is vague. The authors state in line 472 that “[t]his is the policy currently applied in the Ebro basin.” In other words, this is the same as the baseline scenario, right? The only difference being whether you have a drought or not. The way this is described and the subsequent results and discussion beg the question of whether you are conflating institutional and climate scenarios.
Right. The baseline scenario combines normal weather and the policy of institutional cooperation. So, the climate situations are normal climate and drought. Then we have four policies to address drought. Therefore “Institutional Cooperation” is not a scenario, but it is a policy (one of the four policies to deal with drought). The four scenarios combine the drought situation with the four policies to confront drought. This is now clarified in new lines 440-441.

Currently, it is hard to evaluate the policy significance of your findings. The Results section could be significantly improved by clarifying and incorporating some of the issues raised above with regard to the Methods section. Specifically, I would suggest the following:
1. Why not elaborate the results for the alternate institutional scenarios under both Baseline weather and also under drought? Currently, it is impossible to completely separate the effects of institutional and climate factors. So, Table 2 could be split into two tables.

We don’t consider alternate institutional scenarios, but rather four alternative policies to address drought. The baseline is the current policy (institutional cooperation) under normal weather, and we are interested in the outcomes of the four polices to deal with droughts: institutional cooperation [proportional sharing], water markets, and these two by taking into account environmental benefits. A full analysis of possible institutional scenarios under normal weather and also under droughts is a quite difficult task, because any large institutional change is unlikely since institutional changes are incremental, given the institutional path dependency of water management in countries. The analysis of separating the effects of institutional settings and climate factors is beyond the scope of the paper.

2. Given the relatively high dimension of scenarios and sectors to be detailed in the results, you might simplify a few aspects to facilitate interpretation:
a. Why not report results for alternate institutional arrangements relative to the appropriate baseline? That is, give the % change of the variables rather than their levels. This would help the reader understand the significance of institutions on the results.

As indicated in the previous response a full analysis of different institutional arrangements for water management is beyond the scope of the paper. We are not analyzing full changes in the institutions of water management in the Ebro basin, but rather only some incremental policies to deal with droughts, taking into account institutional path dependency.

b. Given that the urban sector is not actually optimized, I would remove it from the table and simply state the urban water use and benefit levels in the text or in the table caption. Then you clarify the trade-off between environmental and agricultural water use as presented in the table.

As indicated in the response to your point 2 (above), we consider that it is important to maintain the urban information in the table because they are the main component of private and social benefits. The reason is the absolute priority of urban use over any other use, and the small share of urban withdrawals over total withdrawals (so no impacts from drought). We prefer to maintain urban information in the table.

3. As far as I can tell, there is no motivation or context or the drought level chosen (40%). Please offer some rationale for that value.

We offer now the justification for the value, by indicating that there have been four severe droughts with a reduction of nearly 40% in basin inflows during the last thirty years (in years 1989, 2002, 2005 and 2012). Additionally, climate change will further reduce basin inflows by 12% during the present century (CEDEX 2017 [68bis]). We include the justification of the 40% level for severe droughts in new lines 435-439.

4. The benefits for the Water Markets scenario appear to be strictly dominated by those from the Environmental Water Markets. Did/can you explain this and its significance?

Right. Water markets are strictly dominated by environmental water markets in both private irrigation benefits and environmental benefits. The reason is that water use in irrigation (and total water use) decreases considerably in environmental water markets, so that the shadow price of water increases. The value of each unit of water traded among irrigators increase (trading 42 M€ in value), and also the value obtained by irrigators from trading with the basin authority (54 M€) buying environmental flows. So finally private irrigation benefits increase somewhat from 483 (water markets) to 489 (env. water markets), and environmental benefits also increase from 719 to 826 M€.

We include now your observation in new lines 558-560.

The data visualization in Figures 2-4 could be improved. Figure 3, in particular, could be better. Why doesn’t this figure use bar charts showing stream flows by scenario for each location? Also, relative values (compared to baseline) would be better. Figures 2 and 4 are difficult to draw inference. Consider simplifying them and/or adding place names to the maps so that they may be related to Figure 3. Also, Figure 4 includes “Moderate” and “Severe” droughts. How are these defined? I see that on line 466 the level of 40% reduction is cited as a severe drought. Is that the level of drought considered in Table 2? There it is just mentions ”drought.” This is quite confusing

Presenting the information in Figures 2-4 for each policy scenario and for each location would be too cumbersome, since we have four policy scenarios and 21 locations, 12 irrigation districts and 9 urban centers (See new Figure 2 (network) and new Figure S2 (map of basin) in Supplementary Materials) . We decided to present the information by watershed board (these are sub-basins which are the units of water management in the basin), which are important for the assessment of the distribution of environmental benefits across the basin (this is stated in new lines 481-483). The same problem for Figure 3 to include bars for four scenarios and 21 locations. We decided to present the stream flows in several locations along the Ebro main stem, showing the impacts of policies along the Ebro main steam.

Following your comment, we have included the locations appearing in new Figure 3 (old Figure 2), in both the new Figure 3 (old Fig. 2) and Figure S1 (your Figure 4).

Moderate and severe drought: In the text of the article we only consider severe drought (-40%). In old line 466 (new line 435) we state the water inflows are reduced by 40% under severe drought. In response to your comment, we now have included additional text (new lines 435-439) to justify this level of inflow reduction, and we indicate “Severe drought” in Table 2. We only include moderate drought in Supplementary Materials, in Figure S1 and Table S1. In both Figure S1 (your Figure 4) and Table S1 of Supplementary Materials, we now indicate at the bottom of the figure and the table that we include additional information for a more moderate drought (-30%) applying the same methodology (new lines 8-10 and 13-14 in Supplementary Materials).

**Reviewer 2**
Comments

This paper presents the development and application of a hydroeconomic model to understand interactions between environmental and human water uses under water scarcity and drought conditions in the Ebro basin, by explicitly accounting for environmental benefits linked to the river ecological status. This addresses an outstanding research gap in water resources systems modelling to support decision making.

Thank you for indicating that the subject of the paper is of great significance to address the research gap in water modeling. The comments you provide are helpful to improve the introduction, the methodology, and the results.

However, although the hydroeconomic modelling is rigorous there are concerns about quality/soundness of the information used to build and apply the model due to lack of detail and transparency about the methods and their limitations. Moreover, there are several aspects related to the written manuscript which should be addressed before the paper can be considered for publication. General comments on each section are provided below and more detailed comments are provided as annotations on the PDF file.

Thank you for your assessment of our work. We will address each and every comment you provide.

The introduction justifies the relevance and novelty of the research. However, the flow of ideas is confusing as it does not follow a clear structure: general issue > state of the art > research gap > aim and objectives. There is a general lack of references which are needed to support the research questions and selected approach.

Following your recommendations, we have changed the introduction by including citations and concrete examples for supporting the research questions and the selected approach (new lines 44-47, 49-53, 57-61, and citations). We indicate now explicitly what is the research gap (new lines 94-103) and the aim and objectives (new lines 110-115) of the paper.

The methods section describes how generally the assessment of environmental benefits is done supported by literature references more as an academic document to teach a subject than a scientific paper, which should not be part of the methods (perhaps summarised as part of the introduction and/or discussion). A methods section should be “recipe” that clearly and concisely describes how the results were obtained in this particular research so that other researchers could follow it to obtain the same results.

Following your indications, we have modified the methods section by reducing the general description of the assessment of environmental benefits (old lines 145-217), which is now condensed in new lines 197-221. Old figure 1 has been eliminated and the explanation of the WUA function has been expanded, with the new text appearing now in new lines 223-269. Also, a new diagram (new figure 1) has been elaborated to aid reader intuition on the procedure followed to estimate environmental benefits in river reaches. The description of the Ebro River basin has been moved from the methods section to a new section before the methods section (following the indications of reviewer 1).

This is somehow achieved from the sub-section “Ebro basin” onwards, although some details are missing which are mentioned in the PDF (e.g. ecosystem services considered and how they were valued – the study uses three valuation levels which are not appropriately justified;

We clarify now that benefits $B_{s}^{eco}$ depend on ecosystem health status $EW_{s}^{eco}$ (driven by streamflow with values from 0 to 1), the length $l_{s}$ of the reach (km), and the economic value of ecosystem services $VE_{s}$ (€/km), as stated in equation 15. The economic valuation of ecosystems has been taken from the range of values found in studies in the literature. Valuation in the literature ranges from 2,000 to 40,000 €/ha of riverbed covered by water [44, 55, 66, 67]. We looked in the studies at valuations of the full range of services provided by water dependent ecosystems. These values are usually given in euros per hectare of riverbed, and they have been converted to euros per kilometer, as explained in new lines 404-407. From the range of values found in the literature, and form the study of ecosystem values in the Ebro by the Spanish Ministry of Environment [55], we have selected three levels of values for $VE_{s}$ which change by the location of the river reach. A low value (0.072 M€/km) for river sections with moderate environmental value in the main steam of the Ebro and in some right bank tributaries, a medium value (0.180 M€/km) for non-mountain Ebro tributaries, and a high value (0.450 M€/km) for mountain river reaches and also for the Ebro Delta.

We now explain in new lines 399-414, how the values of $VE_{s}$ are obtained.

design and justification of the scenarios – the drought scenario is generated reducing 40% water inflows to the system but no justification is given about what are the basis for this).

As indicated above in the response to the comment by reviewer 1, the justification for a severe drought with reductions of 40% in water inflows is that: there have been four severe droughts with a fall around 40% in basin inflows during the last thirty years (in years 1989, 2002, 2005 and 2012). Additionally, climate change will further reduce basin inflows by 12% during the present century (CEDEX 2017 [68bis]). We include the justification of the 40% level for severe droughts in new lines 435-439.

Relevant details (as they help demonstrate the validity/limitations of the modelling outputs) about how the optimisation was performed are missing. What algorithm was used to solve the objective function? Is the source code of the model available?

We indicate now in new lines 429-431 that we have used the GAMS optimization package with the CONOPT 4 solver. We indicate also that the GAMS code of the model is available in Supplementary Materials.

The results and discussion section (which should actually be two separate sections in the paper) does not include any kind of discussion about where the findings of this study sit in relation to previous studies in the field (there is just one citation in this section!) and the wider implications of the approach taken, including its limitations.

Following your suggestion, the results and discussion section has been separated in two sections (Results and Discussion). We indicate now in new lines 626-636 the results and limitations of previous studies in the field of hydroeconomic modeling that include environmental benefits. We describe also in new lines 637-644 the wider implications of our approach, and in new lines 645-654 the limitations in our study.

The conclusions are too long and are not actually a conclusion (i.e. the answer to the question) but a summary of the results. At the beginning of the study, the aim indicates what you set out to do (i.e. the economic and environmental impacts of droughts and water scarcity in the Ebro basin, and the social welfare that can be achieved under alternative water allocation policies considering environmental benefits), but the answer to that does not come through clearly enough to conclude the paper.

We have reduced the length of the conclusion section (from old lines 622-694 to new lines 657-715). We have changed the content of conclusions in order to focus on the aim of the study and clarify the answer, namely the economic and environmental impacts of droughts, and the comparison of social welfare achieved under alternative water allocation policies (new lines 670-692).

Comments in the pdf

Page 1 (old line 21): the last sentence has been changed (new line 21).

Page 2 (old line 41): a reference has been added (new line 41).

Page 2 (old line 42 and old line 44): the paragraph has been reorganized and three citations have been included to support the statements. Also, some concrete examples are included for a better motivation (new lines 42-63).

Page 3 (old line 61): we now indicate in the text (new lines 68-69 that selected notable works supported by hydroeconomic analysis can be found in Ward (2021) [3oct].

Page 4 (old line 78): we have corrected the text (new lines 82-83).

Page 4 (old line 79): we have corrected the text to wet area (new lines 84-85).

Page 4 (old line 87): we indicate now that the response is based on the biophysical information of inundation dynamics in floodplains (new lines 92-93).

Page 4 (old lines 93-95): done (new lines 108-109).

Page 4 (old line 96): the research aim and approach are now clearly stated in lines 110-115.

Page 5 (old line 105): the paragraph has been moved to new lines 94-101.

Page 5 (old line 115): both paragraphs have been shortened (new lines 125-143).

Page 6 (old lines 146 and 147): we include the citation of Momblanch et al. (2016) [23] where the issue is explained (new line 199).

Page 7 (old line 148): the first sentence has been eliminated (new lines 199-200), and the following paragraphs have been shortened following the indications of reviewer 1 (new lines 203-221).

Page 7 (old line 170): this text has been eliminated.

Page 19 (old line 439): we have changed this paragraph to better explain how we calculate the three levels of VE (economic valuation of ecosystems’ services). This is already discussed above in the response to your comment “some details are missing which are mentioned in the PDF (e.g. ecosystem services considered and how they were valued – the study uses three valuation levels which are not appropriately justified;”

See the response above:

“We clarify now that benefits $B_{s}^{eco}$ depend on ecosystem health … …for the Ebro Delta.

We now explain in new lines 399-414, how the values of $VE_{s}$ are obtained.”

Page 20 (old line 465): This is already discussed above in the response to your comment “the drought scenario is generated reducing 40% water inflows to the system but no justification is given about what are the basis for this)”

See the response above:

“the justification for a severe drought with reductions of 40% in water inflows is that… …during the present century (CEDEX 2017 [68bis]). We include the justification of the 40% level for severe droughts in new lines 435-439.”

Page 22 (old line 489): We indicate normal weather to distinguish it from drought weather, because both normal and drought weather are historical (e.g. droughts with 40% inflow reductions in years 1989, 2002, 2005 and 2012). Naming it historical can be confusing.

Page 23 (old line 523): thanks for your comment. The statement is not supported by the data (e.g. data in new Figure 3). Old lines 523 and 524 have been eliminated.

Page 24 (old line 527: in the response to the last comment of reviewer 1 above, we indicate that:

“In the text of the article we only consider severe drought (-40%). In old line 466 (new line 435) we state the water inflows are reduced by 40% under severe drought. In response to your comment, we now have included additional text (new lines 435-439) to justify this level of inflow reduction, and we indicate “Severe drought” in Table 2. We only include moderate drought in Supplementary Materials, in Figure S1 and Table S1. In both Figure S1 (your Figure 4) and Table S1 of Supplementary Materials, we now indicate at the bottom of the figure and the table that we include additional information for a more moderate drought (-30% applying the same methodology) (new lines 8-10 and 13-14 in Supplementary Materials).”

So, we only consider severe drought (-40%) in the text of the paper, and in Supplementary Materials (Table S1 and Figure S1) we indicate that we include additional information for a moderate drought (-30%) applying the same methodology.

Page 24 (old line 529): we have changed old Figure 2 (new Figure 3) and Figure S1, indicating in the legend that these are “Environmental benefits in the basin and in local watersheds (map)”. We also indicate in new lines 481-483 that environmental benefits are displayed by local watershed, because they are the water management units in the basin

Page 25 (old line 564): yes, the environmental water markets policy is described in the methods section (in the Modeling policy scenarios sub-section). Here we only remind some details of the policy.
